# Canine and Phocine Distemper Viruses: Global Spread and Genetic Basis of Jumping Species Barriers

**DOI:** 10.3390/v11100944

**Published:** 2019-10-14

**Authors:** Judith M. Kennedy, J.A. Philip Earle, Shadia Omar, Hani’ah Abdullah, Ole Nielsen, Melody E. Roelke-Parker, S. Louise Cosby

**Affiliations:** 1 Wellcome Wolfson Institute for Experimental Medicine, Queen’s University, Belfast BT9 7BL, UK; judithkennedy22@gmail.com (J.M.K.); omershadia@yahoo.co.uk (S.O.); hanieabdll@yahoo.com (H.A.); 2 Department of Fisheries and Oceans Canada, Winnipeg, Manitoba R3T 2N6, Canada; Ole.Nielsen@dfo-mpo.gc.ca; 3 Leidos Biomedical Research, Inc., National Cancer Institute, Frederick, MD 21702-1201, USA; melody.roelke-parker@nih.gov; 4 Virology Branch, Veterinary Sciences Division, Agri-Food and Biosciences Institute, Belfast BT4 3SD, UK

**Keywords:** canine distemper virus, phocine distemper virus, morbillivirus, global spread, genetic analysis, species barriers, receptors, climate change

## Abstract

Canine distemper virus (CDV) and phocine distemper (PDV) are closely-related members of the *Paramyxoviridae* family, genus *morbillivirus*, in the order *Mononegavirales*. CDV has a broad host range among carnivores. PDV is thought to be derived from CDV through contact between terrestrial carnivores and seals. PDV has caused extensive mortality in Atlantic seals and other marine mammals, and more recently has spread to the North Pacific Ocean. CDV also infects marine carnivores, and there is evidence of *morbillivirus* infection of seals and other species in Antarctica. Recently, CDV has spread to felines and other wildlife species in the Serengeti and South Africa. Some CDV vaccines may also have caused wildlife disease. Changes in the virus haemagglutinin (H) protein, particularly the signaling lymphocyte activation molecule (SLAM) receptor binding site, correlate with adaptation to non-canine hosts. Differences in the phosphoprotein (P) gene sequences between disease and non-disease causing CDV strains may relate to pathogenicity in domestic dogs and wildlife. Of most concern are reports of CDV infection and disease in non-human primates raising the possibility of zoonosis. In this article we review the global occurrence of CDV and PDV, and present both historical and genetic information relating to these viruses crossing species barriers.

## 1. Introduction

Canine distemper virus (CDV) and phocine distemper (PDV) are closely-related members of the *Paramyxoviridae* family, genus *morbillivirus*, in the order *Mononegavirales*. Other members of the genus include the measles virus, the now eradicated virus of cattle, rinderpest, peste des petits ruminant virus of sheep and goats, and the cetacean morbilliviruses. Both CDV and PDV spread in bodily secretions, and transmission is largely through respiratory routes. CDV is highly contagious among susceptible host species, occurs worldwide, and despite vaccination efforts, the number of cases has increased in recent decades [1]. CDV has a broad host range among carnivores, with the infection of species in the family *canidae*; dog, wolf, jackal, coyote, fox, dingo, as well as black-footed ferrets. 

The last wild population of black-footed ferrets was wiped-out in 1985 due to CDV infection [2], and this virus currently threatens the endangered African wild dog species. Of most concern are the reports of CDV infection and disease in non-human primates, raising the possibility of zoonosis [3]. 

PDV is thought to be derived from CDV through contact between terrestrial carnivores and seals. PDV can infect many species of seal and terrestrial carnivores as well, and is evidenced by the transmission of the disease to mink in the immediate vicinity of diseased seals in 1998 in Denmark [4]. Furthermore, CDV from terrestrial hosts has itself caused epidemics among land-locked Baikal seals and Caspian seals (*P. caspica*) since the late 1980s [5,6]. PDV has caused extensive mortality in Atlantic seals, and more recently has spread to the North Pacific Ocean, and there is also serological evidence of morbillivirus infection in seals in Antarctica. In this article we review both the global occurrence of CDV and PDV, and present both historical and genetic information on these viruses crossing species barriers. 

## 2. Morbillivirus Proteins 

Morbilliviruses encode six structural and two non-structural proteins. The RNA genome is within the internal helical nucleocapsid, which incorporates the structural proteins nucleoprotein (N), the phosphoprotein (P) and the large protein (L). This forms a ribonucleoprotein complex together with the RNA-dependent RNA polymerase (RdRp). [7,8]. The L protein activates the RdRp through its interactions with the P and N proteins. The RdRp is responsible for the transcription and replication of the virus genome, and also carries out modifications of the mRNAs post-transcriptionally. The lack of proof-reading ability of the RdRp accounts for the high mutation rate associated with RNA viruses [8]. The virus is enveloped by a lipid bilayer which is derived from the host cell when budding from the plasma membrane. The matrix (M) protein presents a boundary between the nucleocapsid and the envelope, and plays a role in the transcription and budding of the virus [9,10,11]. 

The haemaglutinin protein (H) and the fusion protein (F) are incorporated in the virion envelope [12]. The F protein mediates the fusion between the virus envelope and the cell membrane to allow the entry of the nucleocapsid into the cell cytoplasm. The H protein mediates virus attachment to the cell receptor, and its sequence is highly variable, with often single amino acid changes altering the ability of this virus to infect cells from different species [13]. CDV and PDV, like the measles virus and the other veterinary morbilliviruses, use both the signaling lymphocyte activation molecule (SLAM) and nectin-4 as receptors [14,15,16]. Although evidence is not available for all morbilliviruses, it has been shown in mice and primate models of MeV infection that alveolar macrophages and dendritic cells expressing SLAM are initially infected on virus entry through the respiratory track. Nectin-4 is an epithelial cell basal receptor important for virus release from tissues including the lungs [17,18,19]. The viral P protein is much less variable, but has proven useful in morbillivirus phylogenetic studies. In addition to the P protein, the P gene also encodes two non-structural proteins V and C which modulate the immune response. The V protein has been shown to inhibit the interferon (IFN) system by blocking the Jak/Stat signaling pathway while the C protein acts as a regulator of viral RNA synthesis, thereby acting indirectly to suppress IFN induction. [20,21,22] Therefore, the P gene sequence is an important indicator of virus virulence, and in the now eradicated morbillivirus, rinderpest virus has been found to be important for cross species pathogenicity [23]. 

## 3. Origin of CDV

European dogs were first introduced to the New World by Christopher Columbus (1451–1506) in 1493, but epidemics of unusual mortality in either the European or native dog populations did not occur at this time. Widespread epidemic measles in the Americas closely preceded the first descriptions of CDV-like epizootic outbreaks of disease in dogs, suggesting infection of canines by MeV. Dogs were regularly exposed to measles-ridden human flesh from the Native American population in the New World. However, this origin of CDV from MV cannot be proven. The first reliable report of canine distemper was in Ecuador and Peru, and the earliest definitive reports of canine distemper epidemics in Europe occur after the disease was first described in South America.

Scientists of the time, including Edward Jenner (1749–1823), noted that the disease was entirely unknown in Europe prior to the mid-1700s [24]. 

## 4. Geographical Spread and Species Jumping of PDV and CDV

### 4.1. PDV Spread and Species Susceptibility

Phocine distemper virus was first characterized by us and others as an important pathogen of seals in 1988 when an outbreak caused mass mortality among European harbor seals (*Phoca vitulina vitulina*) in the North Atlantic Ocean [25,26]. Differences between the 1988 and 2002 PDV isolates identified through phylogenetic analysis supported the introduction of a new virus. We previously determined that a PDV isolate from a harbor seal infected during a 2006 outbreak on the US Atlantic coast showed more similarity (99.3% across the genome, to the 1988 isolate, suggesting the possibility of multiple viral lineages circulating in Arctic and Atlantic seal populations [27] (GenBank accession numbers for the 1988, 2002, and 2006 outbreaks viruses are NC_028249, FJ648456, and KY629928, respectively). Atlantic harbor seals were highly susceptible to these three strains of PDV, and suffered large population losses associated with infection [28]. In sympatric species, including gray seals and arctic species, such as harp and hooded (*Cystophora cristata*), sporadic deaths do occur, but at considerably smaller numbers to those seen with harbor seals [29]. In 2000 from April to August, thousands of Caspian seals (*Phoca caspica*) died in the Caspian Sea. This outbreak was confirmed both serologically and by PCR to be CDV and not PDV [30].

### 4.2. PDV in North Pacific Ocean

Serologic surveys before 2000 indicated that Pacific marine mammals had not been exposed to PDV [31,32], and this virus had never been identified as the cause of illness or death in the North Pacific Ocean (NPO). PDV was first confirmed in sea otters in the NPO in 2004 [33], posing the question of whether reductions in sea ice believed to be due to global warming could increase contact between arctic and subarctic marine mammals and lead to viral transmission across the Arctic Ocean. We have recently examined this question using serological and PCR data from samples obtained in the NPO between 2001–2016; Findings will be reported elsewhere [34].

### 4.3. Morbillivirus Spread to the Antarctica

There was a mass mortality event in crab eater seals in 1955 in the Prince Gustav Channel, which is bounded on the west by the Antarctic Peninsula, and on the east by James Ross Island. Animals had lesions similar to CDV, but no serology is available for confirmation [35]. Antibodies to CDV have been reported in leopard and crab eater seals around the Antarctic Peninsula [36], and PDV in Weddell seals from Vestfold Hills, East Antarctica [37]. As Antarctica has no terrestrial carnivores, it was suggested that Greenland sledge dogs, used in Antarctica before effective CDV vaccines were available, may have transmitted the disease to seals. However, Antarctic seals are known to travel further afield to South America, New Zealand, Australia and South Africa, and so they could have become infected in one or more of these locations [38]. More recently we have sampled seals in Antarctica, and also found a high percentage of these to have neutralizing antibody levels when tested against CDV and PDV (unpublished data) [39]. 

### 4.4. Canine Distemper Virus Spread to Felines and Other Wildlife Species

In the last three decades, spill over from viral reservoirs in canines has implications for endangered wildlife, including lions, tigers, leopards, raccoons and pandas [40,41]. Prior to 1991, when an outbreak of the virus caused deaths in captive large cats in separate locations in North America [42], CDV was not thought to cause clinical disease in felines. The same symptoms of CDV infection, i.e., seizures, myoclonus and ataxia, were seen in 1993 in the lion *(Panthera leo*) population from the Serengeti Park (SNP) in Tanzania. Over 85% of the lion population was infected, and 35% died within six months of this infection [43,44,45,46]. Other inhabitants of the SNP were also infected, including the spotted hyena, (*Crocuta crocuta*) and the bat-eared fox (*Otocyon megalotis*) [47,48]. 

A second high-mortality CDV epidemic struck the nearby Ngorongoro Crater lion population in 2001. Serological analyses has indicated that at least five ‘‘silent’’ CDV epidemics swept through the same two lion populations between 1976 and 2006 without clinical signs or measurable mortality [48]. In December 2000 CDV spread through a captive breeding group of African wild dogs (*Lycaon pictus*) in Tanzania, killing 49 of 52 animals within two months, with evidence of this virus continuing to circulate, threatening this population [49,50]. The high density of the domestic dog population to the west of the Serengeti National Park is thought to have allowed CDV to be maintained as a stable infection in this area. Spill over from this population is considered to have resulted in the infection of animals in the Park [51]. As the ‘‘silent’’ CDV epidemics in the Serengeti were more frequent, it has been suggested that a major contributing factor to the extent of adult lion mortalities in the 1994 and 2001 outbreaks was hemoparsitism with *Babesia* during periods of drought [48]. An alternative suggestion by Nikolin et al. [52] is that lions and hyenas clear canine-adapted CDV strains before clinical signs develop, and that virus adaption is required to produce disease.

More recently, CDV outbreaks occurred in several reserves within South Africa in a lion population on a privately owned nature reserve in the Waterberg in December 2015, which resulted in 95 % mortality. This outbreak also infected other carnivore species, resulting in the first reported case of CDV mortality in an endangered brown hyena (*Hyaena brunnea*). Four months later, the devastating effect of CDV was also observed in African wild dog populations of Kruger National Park and Tswalu Kalahari Reserve, South Africa, with the total eradication of two packs [53]. This raises questions with regard to the virulence and pathogenicity of circulating virus strains.

### 4.5. CDV Infection in Non-Human Primates

Four outbreaks of CDV in non-human primates have been reported in the last 30 years. A case of encephalitis in a Japanese macaque (snow monkey) (*Macaca fuscata*) was reported in 1989, and 22 such monkeys in the same group as the diseased monkey had relatively high titers of neutralizing antibody to CDV. The pattern of the antibody titers to CDV and MV closely resembled that of cynomolgus monkeys experimentally inoculated with CDV [54]. In 2006 a large CDV outbreak on a Guangxi breeding farm occurred, where approximately 10,000 animals were infected, and between 5%–30% died. The epidemic was controlled by vaccination [55], but probably due to secondary transmission, two to three years later, an outbreak of CDV in hand-feeding Rhesus monkeys (*Macaca mulatta*) was reported in Beijing, with 20 monkeys presenting with respiratory and neurological symptoms. They displayed anorexia, acute fever, thickening of the footpad and facial rashes. Twelve animals died, which suggested a high virulence of this CDV strain for monkeys. Gross pathological examination showed lesions in the lungs consistent with pneumonia, and diffuse hemorrhage in the CNS consistent with distemper infection. Phylogenetic analysis confirmed that the monkey-adapted CDV belonged to the clade of epidemic CDV types circulating in China [56]. In 2008 in Japan, a CDV outbreak also occurred in cynomolgus monkeys imported from China. Forty six monkeys died from severe pneumonia during a quarantine period. Phylogenic analysis also showed that the isolate from this outbreak, CYN07-dV, was closely related to the CDV outbreaks in China, suggesting continuing chains of CDV infection in monkeys [57].

### 4.6. Evidence for CDV Vaccine Spread to Wildlife

Cases of distemper due to vaccine administration have been documented in dogs globally, but evidence is often inconclusive due to lack of molecular testing in older studies. The propensity of the Rockborn vaccine strain, (which was attenuated from a virus isolated in 1950s Sweden), to revert to virulence, was initially established by Appel in 1978 [58], although the vaccine continued to be used.^.^ In 1982–83, cases of post-vaccinal encephalitis were recorded in dogs in various parts of Britain after the administration of a particular batch of combined distemper/hepatitis vaccine. Detailed investigations of one of these cases revealed that the Rockborn CDV component was responsible, and the vaccine virus was recovered from the brain of the affected dog [59]. In South Africa, clinical signs of a fatal disease resembling those of canine distemper were observed in two groups of captive wild dog pups 13 days after vaccination with a combined vaccine also containing a CDV component [60].

The Rockborn vaccine was supposedly withdrawn from markets after the mid-1990s. However, Demeter et al. and Martella identified Rockborn-like viruses in vaccines currently on the market [61,62]. Vaccine X (also called Vaccine D and Vanguard), a Rockborn-based vaccine, has been used in recent years in Africa. Isolates from vaccinated dogs with distemper in Africa were investigated [63,64]. The isolates were only distantly related to Ondersteport- and Snyder Hill-derived vaccines based on phylogenetic analysis of the H gene of CDV. Vaccine D was more closely related to the isolates, but could still be distinguished from them and showed 99% nucleotide identity to a Hungarian vaccine strain (EF095750) and to a Chinese CDV isolate from a lesser panda (red panda— *Ailurus fulgens*) (AF178039) [64]. Disease in the lesser panda had previously been demonstrated to be vaccine-induced [65]. Pardu et al. [66] also reported a North American isolate from a vaccinated dog with distemper which was related to the lesser panda strain. Vaccine D also grouped in lineage America 2 with many CDVs of wildlife species [64], raising the possibility of a vaccine-derived virus in these species. An alternative explanation is that the virulent Rockborn parent virus infected wildlife in Africa and on other continents. However, the occurrence of highly related viruses in such diverse locations would be consistent with vaccine distribution worldwide, and could account for the wide geographical spread of Rockborn-like viruses, their reversion, and cases of disease in vaccinated animals. 

## 5. Genetic Analysis of Cross Species Infection

### 5.1. Adaptation to Non-Canine Species

Analysis of genetic data of isolates provides a means to help inform our understanding of cross species transmission, as well as the role of specific mutations in virus pathogenicity. Molecular analysis of the H and P genes of isolates from the 1993 Serengeti outbreak were previously made by Carpenter et al. [67], and more recently by Nikolin et al. [52]. These studies indicated genetic clustering within the geographical area rather than within a host species. We have also carried out a sequencing analysis of the H and P genes from five CDV isolates out of four animal species from the Serengeti outbreak in 1993/1994 (Table 1), as well as for comparison the P gene of the Duramine vaccine (Forte Dodge Animal Health, Iowa USA) accession number MN400967.

Sequence comparison was made to all available CDV strains in GenBank, including the sequences of Serengeti isolates described by Nikolin [52], monkey isolates and vaccine strains. Materials and Methods are given in the Supplementary information file. Based upon available sequences, there are five synapomorphic base changes (three silent) in the H gene, and five in the P gene (three silent) which distinguish all the Serengeti isolates from all other viruses in GenBank (Table 2). Phylogenetic trees for the P and H genes were produced for the five Serengeti isolates and selected CDV sequences available in GenBank using Neighbor Joining and Fast Minimum Evolution programs. Total P gene data was only available for some of the viruses used. 

### 5.2. P Gene Sequences

**Figure 1:**
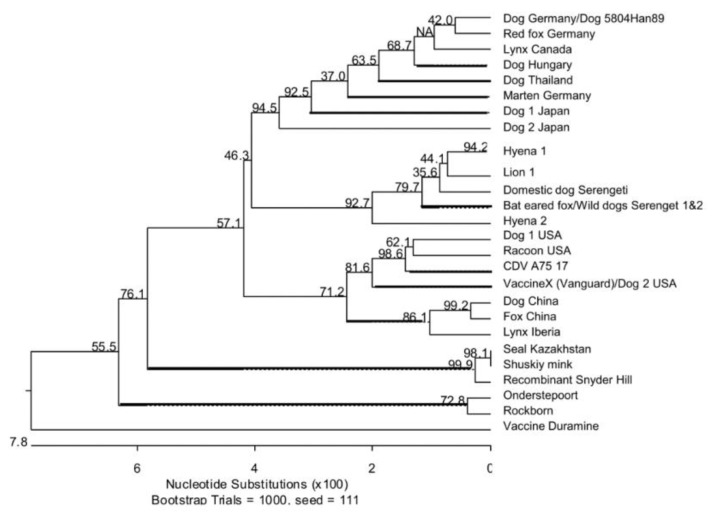
Phylogenetic relationship of CDV strains from the 1993/1994 Serengeti CDV epidemic to other selected strains of CDV available in GenBank based on P gene sequences. Dog Thailand (AB299193.1), Marten Germany (AJ582389.1), Dog 1 Japan (AB294371.1), Dog 2 Japan (007Lm, AB212727.1), Hyena 1 (MN335918), Lion 1(MN335916), Domestic dog Serengeti (MN335914), Bat-eared fox (MN335913), Serengeti wild dog 1 (EU481827), Serengeti wild dog 2 (EU481828), Hyena 2 (MN3359), Dog 1 USA(EU716337.1), Raccoon USA (AY649446.1), CDV A75/17(AF164967.1), Vaccine X (EU072201.1), Dog 2 USA (AY964113.1), Dog China (HQ540292.1), Fox China (HQ540293.1), Iberian lynx (GU001865.1), Seal Kazakhstan (HM046486.1), Shuskiy mink (HM063009.1), Recombinant Snyder Hill (GU138403.1), Dog Germany (J582384.1), Dog 5804Han89 (AY386315.1), Red fox Germany (JN1530301.1), Lynx Canada (FJ240229.1), Dog Hungary (AJ582385Onderstepoort (AF378705.1), Rockborn (AF181446.1), Vaccine Duramine (MN400967). Unrooted neighbor-joining phylogenetic trees were constructed by using the MegAlign version 7.1 package (DNASTAR, www.dnastar.com) with the ClustalW method (www.clustal.org). Percentage bootstrap values, indicating the significance of clusters, are shown.

A phylogenetic tree for the P gene for a selected range of viruses is shown in Figure 1. In line with a high conservation of the P gene there is 95% identity across all CDV strains. The Serengeti viruses form a closely-related and genetically distinct group, and not surprisingly have high identity with Serengeti isolates sequenced in previous studies [52,67]. There is 100% identity of the P sequence for the bat-eared fox with viruses isolated from wild dogs in the Serengeti in 2007, suggesting that this strain has continued to circulate.

Nikolin et al. (51) reported that a single nucleotide substitution at site 134 in both the P and V proteins, which resulted in a nonsynonomous amino acid substitution, encoded glycine (G) in all canine Serengeti isolates and encoded serine (S) in all non-canine Serengeti isolates. This substitution results in a glutamine at site 126 [52] in the C protein.

These changes are also confirmed in the Serengeti isolates reported here (Supplementary Figures S1 and S2). We have also observed a change at site K117Q in the C protein for all of the Serengeti isolates which encodes a glutamine, whereas all other viruses have a lysine. In common with a German fox, Hyena 1 shows a change to valine from the consensus glutamic acid at residue 115. Both the dog and lion isolates have a leucine at this site, while the bat-eared fox has an alanine (Supplementary Figure S2).

We have also noted two changes that occur in the P protein between CDV disease-causing (including monkey) and vaccine strains at sites (D56V, T135A) with the latter residue in each case being associated with disease. However, at both these sites Vaccine X (Vaccine D, Vanguard) has the ‘disease-associated amino acid’ and the seal Kazakhstan and Shuskiy mink CDV viruses as well as PDV stains have the ‘vaccine amino acid’ (Supplementary Figure S1). It is possible that these residues are important in adaptation to seals, and differ to those related to pathogenicity in land mammals. 

The data suggest that on some occasions the virus may have acquired mutations in the P/V/C sequences related to increased virulence on passage through various host species, but experimental evidence will be required to substantiate this. The presence of residues in all the Serengeti isolates, which the analysis indicates may be important markers of virus virulence in wildlife, are also present in Vaccine X. These sites as a prediction of pathogenicity are not the same as those suggested in experimental studies in ferrets [68], as the potential virulence-associated changes in the latter are not reflected in wildlife CDV strains. Although a lion CDV strain was previously shown to be virulent in ferrets [69], this species is highly sensitive to CDV, and therefore unlikely to reflect pathogenicity in lions and other wildlife. Unfortunately, confirmation of new virulence-related mutations by comparing the P/V/C sequences of the Serengeti isolates with those of viruses giving rise to the ‘silent’ infections in lions, cannot be made. 

Isolates from these animals are not available, as CDV sera positivity was determined retrospectively when blood samples were taken from lions during collaring.

### 5.3. H gene Sequences

**Figure 2:**
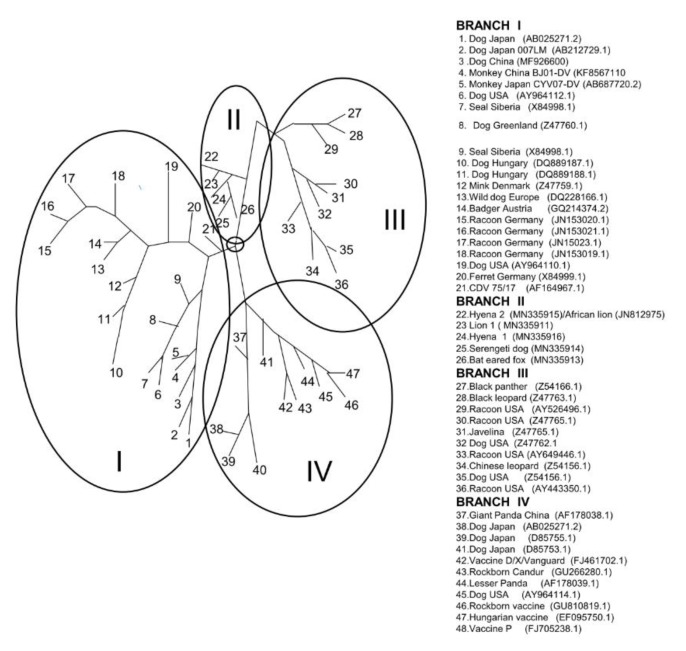
Phylogenetic relationship of CDV strains from the 1993/1994 Serengeti epidemic to related viruses based on H gene sequences. Circles represent branches (I, II, III and IV) from a common node (black circle). Accession numbers are given for isolates in the figure key. Associated references where available for viruses as numbered in the key: 1, 37. Mochizuki et. al. [72]; 2. Lan et al. [73]; 3. Qiu et al [56]; 4. Feng et al. [71]; 5. Sakai et al. [57]; 6, 8, 12, 29, 30, 31, 34. Bolt et al. [74]; 7. 20. Mamaev et al. [5]; 8, 9, 10. 11 Demeter et al. [75]; 12. Martella et al. [62]; 14,15,16,17 18. Nikolin et al. [76]; 19, 43 Pardu et al. [66]; 21, Weiderkehr et al (unpublished); 22(Hyena)-24 Current report; 22 (lion). Nikolin et al. [52], 33. Harder et al. [77]; 28, 32, 35. Lednicky et al. [78]; 38, 39. Iwatsuki et al. [79]; 40. Woma et al. [64]; 41, 44. Martella et al. [80] 29: 1222–1227; 46. Chulakasian et al. [81]. Unrooted neighbor-joining phylogenetic trees were constructed by using the MegAlign version 7.1 package (DNASTAR, www.dnastar.com) with the ClustalW method (www.clustal.org).

The H gene is much more variable than the P gene, with only 91% identity across CDV strains. A phylogenetic tree for selected isolates is shown in Figure 2. H amino acid changes were compared for selected strains. The substitutions P327L, K375Q, A/359/S, Q/402/P and I527V in the H protein distinguish most Serengeti isolates (ours and those of Nikolin et al. [52]) from all other CDV strains. However, Hyena 1 (MN335912) does not have the I527V substitution, while a V at this site also occurs in PDV/USA. The dog (MN335909) and bat-eared fox (MN335908) isolates also have a unique D307M substitution. Two substitutions I541P and P52I are unique to the bat-eared fox. Hyena 2, which was the earliest isolate, shares a Y549H change with Lion 1 and the lion and hyena isolates from the study by Nikolin et al. [52]. This change to histidine is also shared by the Ondersteport and Convac vaccine strains, as well as the Caspian PDV isolate. Sites 530 and 549 were suggested to be associated with the spread to non-canine hosts by McCarthy et al. [70].

Phylogenetic analysis of the full-length H gene showed that Monkey CYN07-dV [58] and Monkey-BJ01-DV were highly related to other CDV strains obtained from species of the *Canidae* family in China during this time period. The monkey CDV strains (Monkey-BJ01-DV, CYN07-dV, Monkey-KM-01) possess a number of amino acid-specific substitutions (E276V, Q392R and D435Y) compared to the H protein of CDV occurring in other animals for the same period [57,71]. An additional change from I542F in these monkey strains is also noted from our current analysis. As expected there are many substitutions for PDV compared to CDV strains. Alignment of selected strains including monkey and PDV isolates is shown in Supplementary Figure S3. 

## 6. Virus Adaption to Cell Entry Receptors

The majority of CDV strains isolated from *Canidae* have tyrosine at site 549, whereas CDV strains from other carnivore families mostly have histidine [76].The specificity with which CDV-H interacts with SLAM and its potential as a determinant of host range have been investigated [71,76,82,83,84,85,86]. Amino acid residues Y525, D526 and R529 of CDV-H have been identified by site-directed mutagenesis to interact with SLAM [85]. Amino acid 527 and 549 have also been studied, and it is thought that these are important determinants of infectivity in carnivores [70]. Both 530 and 549 fall into the receptor-binding domain located on propeller β-sheet 5 of the CDV-H protein [87]. 

Specific sites in the H protein of all morbilliviruses are conserved to facilitate efficient SLAM binding [71]. A study by Nikolin et al. [76] showed changes in the SLAM receptor binding site on H with adaption to non-canine species and correlated with functional receptor binding assays. Comparison in the current analysis of the H SLAM binding site of all available strains, including monkey and PDV isolates, shows that at this site all the Serengeti isolates have a valine rather than isoleucine found in the other CDV stains. At residue 530, hyena 1 has a glycine (in common with some other dog strains and monkey-adapted CDV) rather than aspartic acid in the other Serengeti isolates. At amino acid 327 the Serengeti isolates all have leucine rather than aspartic acid. At residue 375 all Serengeti isolates have a glutamine rather than lysine (Supplementary Figure S3). 

Comparison of the amino acid sequences of the entire H binding site in SLAM among various carnivores shows a high similarity among residues from the *Canidae* species.

**Figure 3:**
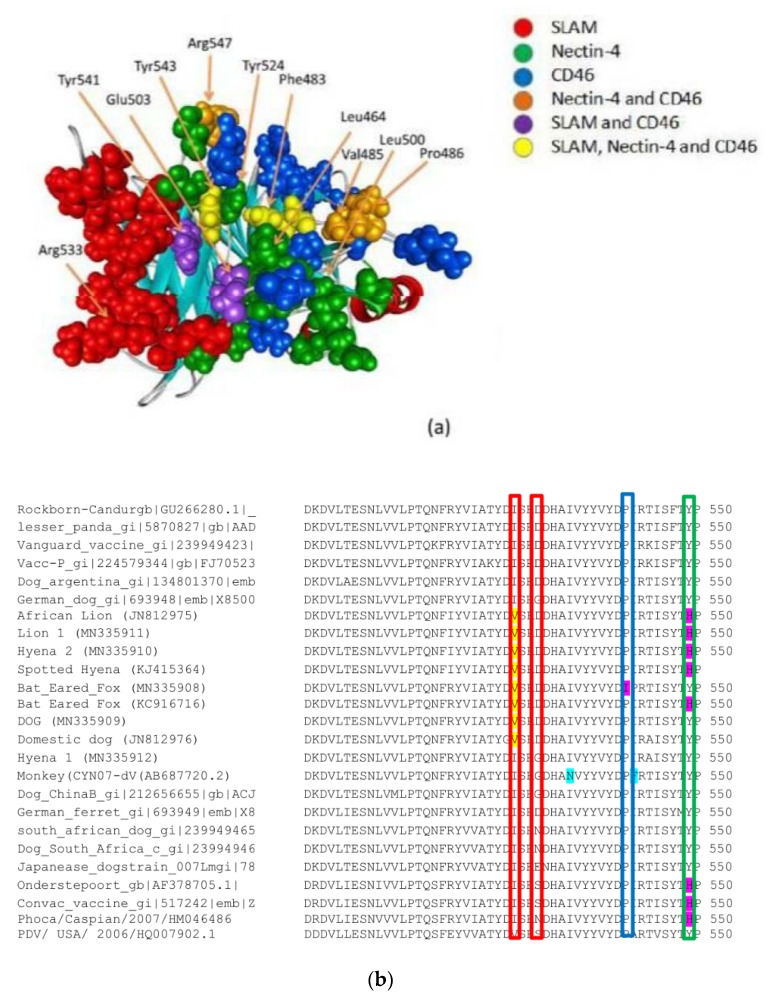
H protein at Receptor binding site. (**a**) Distribution of amino acid residues on morbillivirus haemagglutinin protein, showing significant attraction to the signaling lymphocyte activation molecule (SLAM), nectin-4 and for MeV vaccine strains CD46 (modified from Fenggi et al. [88]. (**b**) Alignment of selected canine distemper virus (CDV) H and phocine distemper (PDV) H amino acid sequences at residues 501–550 in the receptor binding site. Boxes indicate: Red, residues critical for SLAM binding; blue, residue critical for Nectin-4 binding; green, site suggested by McCarthy et al. [70] to be associated with spread to non-canine host. Highlighted residues: Yellow, shared by the Serengeti strains; red, consensus sequence; magenta, differences from the consensus; turquoise specific to monkey virus. Substitutions in PDV have not been highlighted.

This suggests a similar sensitivity to CDV among animals in this group [84]. In contrast, when comparing *Felidae* to *Canidae*, several residue differences were identified that ultimately led to electric charge differences in the SLAM interface of feline species [85]. CDV strains that are well adapted to bind to dog SLAM receptors may therefore be less adapted to bind to SLAM receptors from non-canine hosts. Changes at these sites were observed in our hyena 1, lion and bat-eared fox isolates, while the earlier isolate, hyena 2, maintained the residues associated with CDV infection in dogs (Figure 3 and Supplementary Figure S3). 

Four major sites in the CDV H sequence (amino acids 479, 480 and 493) in the binding region for the basal epithelial receptor nectin-4 for the measles virus [13,89] and CDV and PDV [14,90] were conserved in all viruses (Supplementary Figure S3), as previously reported for CDV strains [52]. However, at 541, which has been shown to be one of the critical residues for nectin-4 binding [89], the bat-eared fox (MN335908) has an isoleucine rather than a proline found in all other isolates (Figure 3 and Supplementary Figure S3). 

## 7. Vaccine Infection of Wildlife

Based on P gene analysis, the majority of vaccine viruses including Onderstepoort, Rockborn and our sequence for Duramine vaccine (MN400967, confirmed to be based on the Onderstepoort vaccine strain) form a distinct cluster (Figure 1). The exception to this is Vaccine X (EU072201.1, alternative names Vaccine D, Vanguard vaccine). From our analysis the Vaccine X P gene shares 100% identity with the Hungarian vaccine, as well as panda and dog isolates from vaccinated animals with distemper, including USA dog isolate (AY964113.1) [71]. These relationships to Vaccine D, as previously discussed, have also been noted by Woma et al [64]. Further isolates from vaccinated dogs with clinical signs have high identity with Vaccine X. These include a virus isolated in Japan from the lymph node (AB212727.1) of a vaccinated dog with distemper [73]. These common P sequences include the D56V and T135A substitutions, suggested in our current analysis to be associated with disease. 

The Rockborn Candur strain also shows 100% identity with the virus from the vaccinated lesser panda across the entire H gene sequence (Supplementary Figure S3) correlating with the original report [65] that the vaccine strain itself caused the disease. 

While the Japanese virus has been placed in the Asia 2 lineage, and the Rockborn-based viruses are grouped within the North American-2 lineage of CDV on the basis of their H protein [64,66], the sequence similarities in the P gene may reflect that they are all vaccine viruses which have acquired common reversion mutations to virulence. 

## 8. Potential for CDV to Infect Humans

The potential for human infection by CDV was raised following the reports that CDV can infect monkeys with fatal consequences [54,55,56,57]. The CDV monkey-BJ01-DV strain was shown to efficiently use both monkey and dog-origin SLAM to infect and replicate in host cells [71,91]. Sequence analyses carried out by Bieringer et al. [83] indicated that only one adaptive mutation in the H-protein at position 540 (D540G) was required for adaptation to human SLAM. Sakai et al. have reported that P541S and R519S are also important for adaption to human cells [91]. Structural modeling indicated that this adaptive mutation in H reflects the sequence alteration from canine to human SLAM at position 70 and 71 from Pro to Leu (P70L) and Gly to Glu (G71E), and compensates for the gain of a negative charge in the human SLAM molecule. This analysis indicates that only a minimal alteration is required for human adaptation [83].

The monkey-derived CDV was also found to use Nectin-4, and so makes use of immune and epithelial receptors of humans, monkey and canines [91]. It is also speculated that CDV may have previously infected humans, as a CUB analysis carried out of proteins of modern CDV strains revealed that the relative adaptiveness of codon usage is uniformly highest for the human pattern. Retention of the CUB pattern in a new species suggests that the selection pressure to adapt to the new species is low. The significance of the human pattern could also indicate that CDV previously infected humans [24]. 

At present, measles vaccination of humans provides at least partial protection against a potential human-adapted CDV gaining hold in the population, as morbilliviruses are monoserotypic and antibody induced to one member has a degree of cross-neutralization [3]. Measles vaccine has been successfully used in dogs to protect against CDV infection [92,93]. However for humans a more CDV-specific vaccine may be required, as this measles vaccine has been shown to give only partial protection in an experimental CDV primate model [94].

## 9. Conclusions

It is speculated that CDV may have originated by the infection of dogs by MV during human epidemics in the New World. It is generally considered that CDV gave rise to the emergence of PDV in seals and other marine mammals while retaining the ability to cause disease in these species without adaptation being necessary. Both CDV and PDV have spread globally, with CDV crossing into many species, including felines and non-human primates. These species jumps have been facilitated particularly by mutations in the virus H protein at the SLAM receptor binding site. However, no study has identified H protein mutations that contributed to CDV infection to non-human primates, suggesting that they may not be required.

Increased virulence of CDV for some species may have occurred due to mutations in the P and other genes. On some occasions Rockborn-based vaccines may have reverted and caused disease in domestic dogs and wildlife. The potential for CDV and PDV to infect humans may require few amino acid changes. Prevention of the spread of such a zoonotic virus in the human population will only be held in check by maintaining high herd immunity to MV.

## Figures and Tables

**Table 1:** Origin of canine distemper virus (CDV) isolates.

*Sample Id	Species of Origin	Tissue Isolated From	Date Collected	Accession No H Gene	Accession No. P Gene
CCR- 71	Hyena 2	Brain	23/12/93	MN335910	MN335915
CCR- 111	Hyena 1	Brain	03/07/94	MN335912	MN335918
PLE- 6411	Lion 1	Lung	18/11/94	MN335911	MN335916
OME- 81	Bat eared Fox	Lymph node	16/07/94	MN335908	MN335913
A94-11/15	Domestic dog	Brain	9/9/1994	MN335909	MN335914

* The field IDs of these animals (in order) are: 93-063 Ccr, 94-179 Ccr, 94-188 Ple, 94-200 Ome, and CFA-51.

**Table 2:** Synapomorphic Changes in Serengeti Isolates.

Gene	NT Number	Base Change	AA Number and Change
H gene	8086	T→C	Silent
	8156	A→C	Silent
	8202	A→C	Pro/327/Leu
	8316	C→A	Lys/375/Glu
	8321	T→C	Silent
P gene	2172	A→C	Glut/124/Ala
	2320	C→T	Silent
	2456	T→G	Silent
	2483	T→A	Silent
	2512	C→T	Ser/236/Leu

## Supplementary Materials

The following are available online at https://www.mdpi.com/1999-4915/11/10/944/s1, Figure S1: Alignment of CDV P/V amino acid sequences 1 to 250 for selected viruses, Figure S2: Alignment of CDV C amino acid sequences, Figure S3: Alignment of CDV H amino acid sequences.
