# Supplementary material for: Canine and Phocine Distemper Viruses: Global Spread and Genetic Basis of Jumping Species Barriers
Source: Viruses. 2019 Oct 14;11(10):944. doi: 10.3390/v11100944 (PMC6833027; doi:10.3390/v11100944)
Supplement: Supplementary file 1 [file viruses-11-00944-s001.pdf]

Supplementary Information

MATERIALS AND METHODS

Cells

Vero.Dog SLAMtag (VDS) cells were grown in Dulbecco's modified Eagle's medium (Invitrogen, California, USA) supplemented with 5% fetal bovine serum. Tissues were collected between 1993 and 1994 from 4 different species, a lion, 2 hyenas, a bat eared fox and a domestic dog which died of CDV infection (Table 1). Samples were obtained from wild animals in the Serengeti Park in Africa which died due to natural infection. Tissues were taken at post mortem. Further details of these samples are given in reference 7. Viruses were isolated by inoculating homogenised tissue onto VDS cells which gave rise to syncytia formation.

Reverse Transcription-PCR and DNA sequencing

Total RNA was extracted from infected cells using TRIzol reagent (Invitrogen, California, USA). cDNA synthesis was carried out using oligo-dT primers and the Superscript first strand synthesis kit (Invitrogen, California, USA). PCR was performed using the High Fidelity Taq kit (Invitrogen, California, USA). RT-PCR was carried out on the first passage with a range of primers designed to conserved regions of the H and P genes.

H, P and F gene primer sequences for RT-PCR and sequencing were either previously published (1, 15, 16, 17) or designed for this study. Primer sequences for successful primer sets are given in Table 2**.** DNA sequencing was performed using a Bigdye 3.1 Terminator Cycle sequencing kit (Applied Biosystems, California USA). Completed PCR products were sent to the Genomics Core Facility, Queens University Belfast for chromatographic preparation.

Despite repeated RNA preparations and RT-PCR reactions with additional primer sets we were unable to obtain the total sequence for viruses except for the earliest isolate Hyena 2. This suggests primer mismatch to sequences in later isolates. For the H gene, contigs corresponding to 2,008 nt for the Hyena 2 starting at the end of the F gene and covering the entire H gene; 939 nt for Hyena 1, 948 nt for the lion, 936 nt for the bat eared fox and 1,704 nt for Duramine vaccine were obtained. For the P gene 419 nt for Hyena1, 1613 nt for Hyena 2, 415 nt for the dog, 378 nt for the bat eared fox and 680 nt for Duramine vaccine were obtained. The PCR products were sequenced (submitted to GenBank) and aligned with all CDV sequences previously published in GenBank.

Phylogenetic Analysis

Nucleotide alignments were made using the NCBI BLAST programme. Unrooted neighbour joining phylogenetic and fast minimum evolution trees were constructed using DNASTAR software (Version 7.1) Megalign package with the CLUSTAL W method.

Supplementary Figure 1

Alignment of CDV P/V amino acid sequences 1 to 250 for selected viruses. Highlighted residues: Yellow- synapomorphies shared by the Serengeti strains; Magenta- differences between Nikolin et al (ref 30) and isolates reported in the current study; Red consensus; Turquoise-residues associated with disease in wildlife: turquoise; Green-residues associated with vaccine/attenuated strains

Dog (MN335914)	--	50
Domestic dog (KJ415358)	MAEEQAYHVSKGLECLKTLRENPPDIKEIQEVSSIRDHTRNPGQENGTTS	50
Bat eared fox (MN335913)	--	50
Bat eared fox (KJ415357)	MAEEQAYHVSKGLECLKTLRENPPDIKEIQEVSSIRDHTRNPGQENGTTS	50
Lion1 (MN335916)	--	50
African Lion (KJ415362)	MAEEQAYHVSKGLECLKTLRENPPDIKEIQEVSSIRDHTRNPGQENGTTS	50
Hyena1 (MN335918)	--	50
Hyena2 (MN335915)	--	50
Spotted hyena (KU578258)	MAEEQAYHVSKGLECLKTLRENPPDIKEIQEVSSIRDHTRNPGQENGTTS	50
African_wild_dog_gi|169218409|	--	50
dog_genbank_gi|4204842|gb|U537	--	50
007japanease_dog_isolategi|787	--	50
dog_Japan_gi|163838503|dbj|AB2	--	50
Marten_German_gi|34576254|emb|	--	50
Iberian_lynxes_gi|299931630|gb	--	50
German_dog2_gb|AY386315.1|	MAEEQAYHVSKGLECLKALRENPPDLEEIQEVSSIRDQTRNPGQENGTAS	50
Fox_china_gb|HQ540293.1|_1694-	MAEEQAYHVNKGLECLKALRENPSDIEEIQEVSSIRDQTRNPGQEDGTAS	50

Dog_china_gb|HQ540292.1|_1694-	MAEEQAYHVNKGLECLKALRENPPDIEEIQEVSSIRDQTRNPGQEDGTAS 50
VaccineX_gi|158702386|gb|EU072	MAEEQAYHVSKGLECLKALRENPPDIEEIQEVSSIRDQTRNPGQENGTAS 50
CDV_A75/17_gb|AF164967.1|_1801	MAEEQAYHVSKGLECLKALRENPPDIEEIQEVSSIRDQTRNPSQANGTAS 50
Dog_USA_gb|EU716337.1|_1801-33	MAEEQAYHVSKGLECLKALRENPPDIEEIQEVSSIRDQTRNPSQANGTAS 50
Onderstepoort_gb|AF378705.1|_1	MAEEQAYHVSKGLECLKALRENPPDIEEIQEVSSLRDQTCNPGQENGTTG 50
Rockborn_gb|AF181446.1|	MAEEQAYHVSKGLECLKALRENPPDIEEIQEVSSIRDQTRNPSQANGTAS 50
Recombinant_Snyder_Hill GU138403	MAEEQAYHVSKGLECLKALRENPPDIEEIQEVSSIRYQTCNPGQENGTTG 50
Racoon_usa_gb|AY649446.1|_1801	MAEEQAYHVSKGLECLKALRENPPDIEEIQEVSSIRDQTRNPSQANGTAS 50
Shuskiy_mink_gb|HM063009.1|_18	MAEEQAYHVSKGLECLKALRENPPDIEEIQEVSSIRYQTCNPGQENGTTG 50
Monkey CYN07-dV (AB687720.2)	MAEEQAYHVNKGLECLKALRENPPDIEEIQEVSSIRDQTRNPGQENGTAS 50
Phoca/Caspian/2007HM046486	MAEEQAYHVSKGLECLKALRENPPDIEEIQEVSSIRYQTCNPGQENGTTG 50

```
Dog (MN335914)                         ------------------------------------------------100
Domestic dog (KJ415358)             MQEEEVSQDLDESHEPAEGSNYVGHVLQNNPGCGESNTALVEAEQPAKDD 100
Bat eared fox (MN335913)              -------------------------------------------------100
Bat eared fox (KJ415357)            MQEEEVPQDLDESHEPAEGSNYVGHVLQNNPGCGESNTALVEAEQPAKDD 100
Lion1 (MN335916)                      -------------------------------------------------100
African Lion (KJ415362)             MQEEEVSQDLDESHEPAEGSNYVGHVLQNNPGCGESNTALVEAEQPAKDD 100
Hyena1 (MN335918)                   ---------------------------------------------------100
Hyena2 (MN335915)                   =--------------------------------------------------100
Spotted hyena (KU578258)            MQEEEVSQDLDESHEPAEGSNYVGHVLQNRPGCGESNTALVEAEQPAKDD 100
African_wild_dog_gi|169218409|         ------------------------------------------------100
dog_genbank_gi|4204842|gb|U537         ------------------------------------------------100
007japanease_dog_isolategi|787         ------------------------------------------------100
dog_Japan_gi|163838503|dbj|AB2         ------------------------------------------------100
Marten_German_gi|34576254|emb|         ------------------------------------------------100
Iberian_lynxes_gi|299931630|gb         ----------------------------------------------- 100
German_dog2_gb|AY386315.1           MQEEEVSQDLDESHEPAKGSNYVGHVLQNNPGSGKGNTALVEAEQPPGPG 100
Fox_china_gb|HQ540293.1|_1694-      MQEEEVSQDLDESHEPAKGSNYVGHVLQNNPGCGESNTALVEAEQPAKDD 100
```

Dog_china_gb|HQ540292.1|_1694-	MQEEEVSQDLDESHEPAKGSNYVGHVLQNNPGCGESNTALVEAEQPAKDD	100
Vaccine X_gi|158702386|gb|EU072	MQEEEVSQDLDESHEPAKGSNYVGHVLQNNPGCGESNTALVEAEQPAKDD	100
CDV_A75/17_gb|AF164967.1|_1801	MQEEEVSQDLDESHEPAKGSNYVGHVLQNNPGCGESNTALVEAEQPAKDD	100
Dog_USA_gb|EU716337.1|_1801-33	MQEEEVSQDLDESHEPAKGSNYVGHVLQNNPGCGESNTALVEAEQPAKDD	100
Onderstepoort_gb|AF378705.1|_1	MQEEEDSQNLDESHEPTKGSNYVGHVPQNNPGCGERNTALVEAEQPPKED	100
Rockborn_gb|AF181446.1|	MQEEEVSQDLDESHEPAKGSNYVGHVLQNNPGCGESNTALVEAEQPAKDD	100
Recombinant Snyder_Hill GU138403	MQEEEDSQNLDESHEPTKGSNYVGHVLQNNPGCGESNSALVEAEQLPKED	100
Racoon_usa_gb|AY649446.1|_1801	MQEEEVSQDLDESHEPAKGSNYVGHVLQNNPGSGESNTALVEAEQPAKDD	100
Shuskiy_mink_gb|HM063009.1|_	MQEEEDSQNLDESHEPTKGSNYVGHVLQNNPGCGESNSALVEAEQLPKED	100
Monkey CYN07-dV (AB687720.2)	MQEEEVSQDLDESHEPTKGSNYLGHVLQNNPGCGESNTALVEAEQPAKDD	100
Phoca/Caspian/2007HM046486	MQEEEDSQNLDESHEPTKGSNYVGHVLQNNPGCGESNSALVEAEQLPKED	100

```
Dog (MN335914)                      -------------------EFIIADADSLVVPAGAVSNRGFERGEGSIDD 150
Domestic dog (KJ415358)             IQPGPGIRCYHVYDHSGEEVKGIADADSLVVPAGAVSNRGFERGEGSIDD 150
Bat eared fox (MN335913)            --------------------LRIADADSLVVPAGAVSNRGFERGEGSIDD 150
Bat eared fox (KJ415357)            IQPGPGIRCYYVYDHSGEEVKGIADADSLVVPAGAVSNRGFERGEGSIDG 150
Lion1 (MN335916)                    --------------------LLIADADSLVVPASAVSNRGFERGEGSIDD 150
African Lion (KJ415362)             IQPGPGIRCYHVYDHSGEEVKGIADADSLVVPASAVSNRGFERGEGSIDD 150
Hyena1 (MN335918)                   -------------------IKGIADADSLVVPASAVSNRGFERGEGSIDD 150
Hyena2 (MN335915)                   IQPGPGIRCYHVYDHSGEEVKGIADADSLVVPASAVSNRGFERGEGSIDD 150
Spotted hyena (KU578258)            IQPGPGIRCYHVYDHSGEEVKGIAGADSLVVPASAVSNRGFERGEGSIDD 150
African_wild_dog_gi|169218409|      ------------------EVKGIADADSLVVPAGAVSNRGFERGEGSIDD 150
dog_genbank_gi|4204842|gb|U537      ------------------EVKGIADADSLVVPAGAVSNRGFERGEGSIDD 150
007japanease_dog_isolategi|787      -----------VYDHSGEEVKGIEDADSLVVPAGAISNRGFERGEGSIDD 150
dog_Japan_gi|163838503|dbj|AB2      ------------------EVKGIEDADSLVVPAGAVSNRGFERGEGSLDD 150
Marten_German_gi|34576254|emb|      ------------------EVKGIEDADSLVVPAGAVSNRGFEGGEGSLDD 150
Iberian_lynxes_gi|299931630|gb      --PGPGIRCYHVYDHSGEEVKGIEDADSLVVPAGAVSNRGFERGEGSLDD 150
German_dog2_gb|AY386315.1|          IQPGPGIRCYHVYDHSGEEVKGIEDADSLVVPAGAVSNRGFERGEGSLDD 150
German_dog1_gi|34576244|emb|AJ      ------------------EVKGIEDADSLVVPAGAVSNRGFERGEGSLDD 150
German_red_fox_gi|341626873|gb      ------------------EVKGIEDADSLVVPAGAVSNRGFERGEGSLDD 150
lynx_Canada_gi|229893628|gb|FJ      ------------------EVKGIEDADSLVVPAGAVSNRGFERGEGSLDD 150
Dog_Hungary_gi|34576246|emb|AJ      ------------------EVKGIEDADSLVVPAGAVSNRGFERGEGSLDD 150
```

dog_Thailand_gi|302128145|dbj|	-------------------EVKGIEDADSLVVPAGAVSNRGFERGEGSLDD	150
fox_china_gb|HQ540293.1|_1694-	VQPGPGIRCYHVYDHSGEEVKGIEDADSLVVPAGAVSNRGFERGEGSLDD	150
Dog_china_gb|HQ540292.1|_1694-	VQPGPGIRCYHVYDHSGEEVKGIEDADSLVVPAGAVSNRGFERGEGSLDD	150
Vaccine X_gi|158702386|gb|EU072	IQPRPGIRCYHVYDHSGEEVKGIEDADSLVVPAGAVSNRGFERGEGSLDD	150
CDV_A75/17_gb|AF164967.1|_1801	IQPGPGIRCYHVYDHSGEEVKGIEDADSLVVPAGAVSNRGFERGEGSLDD	150
Dog_USA_gb|EU716337.1|_1801-33	IQPGPGIRCYHVYDHSGEEVKGIKDAGSLVVPAGAVSNREFERGEGSLDD	150
racoon_usa_gb|AY649446.1|_1801	IQPGPGIRCYHVYDHSGEEVKGIEDADSLVVPAGAVSNRGFERGEGSLDD	150
Onderstepoort_gb|AF378705.1|_1	IQPGPGIRCDHVYDHSGEEVKGIEDADSLVVPAGTVGNRGFERGEGSLDD	150
Duramine	IQPGPGIRCDHVYDHSGEEVKGIEDADSLVVPAGTVGNRGFERGEGSLDD	150
Rockborn_gb|AF181446.1|	IQPGPGIRCDHVYDHSGEEVKGIEDADSLVVPAGTVGNRGFERGEGSLDD	150
recombinant_Snyder_Hill GU138403	IQPGPGIRCYHVYDHSGEEVKGIEDADSLVVPAGTVGNRGFESGEGSPDD	150
Shuskiy_mink_gb|HM063009.1|_18	IQPGPGIRCYHVYDHSGEEVKGIEDADSLVVPAGTVGNRGFESGEGSPDD	150
seal_Kazakhstan_gb|HM046486.1|	IQPGPGIRCYHVYDHSGEEVKGIEDADSLVVPAGTVGNRGFESGEGSPDD	150
Monkey CYN07-dV (AB687720.2)	IQPGPGIRCYHVYDHSGEEVKGIEDADSLVVPAGAVSNRGFERGEGSPDD	150
Phoca/Caspian/2007HM046486	IQPGPGIRCYHVYDHSGEEVKGIEDADSLVVPAGTVGNRGFESGEGSPDD	150

Dog (MN335914)	STEDSGEDYSEGNASSNWGYSFGLKPDRAADVSMLMEEELSALLRTSRNV 200
Domestic dog (KJ415358)	STEDSGEDYSEGNASSNWGYSFGLKPDRAADVSMLMEEELSALLRTSRNV 200
Bat earred_fox (MN335913)	STEDSGEDYSEGNASSNWGYSFGLKPDRAADVSMLMEEELSALLRTSRNV 200
Bat eared fox (KJ415357)	STEDSGEDYSEGNASSNWGYSFGLKPDRAADVSMLMEEELSALLRTSRNV 200
African Lion (KJ415362)	STEDSGEDYSEGNASSNWGYSFGLKPDRAADVSMLMEEELSALLRTSRNV 200
Lion1 (MN335916)	STEDSGEDYSEGNASSNWGYSFGLKPDRAADVSMLMEEELSALLRTSRNV 200
Hyena1 (MN335918)	STEDSGEDYSEGNASSNWGYSFGLKPDRAADVSMLMEEELSALLRTSRNV 200
Hyena2 (MN335915)	STEDSGEDYSEGNASSNWGYSFGLKPDRAADVSMLMEEELSALLRTSRNV 200
Spotted hyena (KU578258)	STEDSGEDYSEGNASSNWGYSFGLKPDRAADVSMLMEEELSALLRTSRNV 200
African_wild_dog_gi|169218409|	STEDSGEDYSEGNASSNWGYSFGLKPDRAADVSMLMEEELSALLRTSRNV 200
dog_genbank_gi|4204842|gb|U537	STEDSGEDYSEGNASSNWGYSFGLKPDRAADVSMLMEEELSALLRTSRNV 200
007japanease_dog_isolategi|787	STEDSGEDYSEGNASSNWGYSFGLKPDRAADVSMLMEEELSALLRTSRNV 200
dog_Japan_gi|163838503|dbj|AB2	STEDSGEDYSEGNASSNWGYSFGLKPDRAADVSMLMEEELSALLRTSRNV 200
Marten_German_gi|34576254|emb|	STEDSGEDYSEGNASSNWGYSFGLKPDRAADVSMLMEEELSALLRTSRNV 200
Iberian_lynxes_gi|299931630|gb	STEDSGEDYSEGNASSNWGYSFGLKPDRAADVSMLMEEELSALLKTSRNG 200
German_dog2_gb|AY386315.1|	STEDSGEDYSEGNASSNWGYSFGLKPDRAADVSMLMEEELSALLKTSRNV 200
German_dog1_gi|34576244|emb|AJ	STEDSGEDYSEGNASSNWGYSFGLKPDRAADVSMLMEEELSALLKTSRNV 200
German_red_fox_gi|341626873|gb	STEDSGEDYSEGNASSNWGYSFGLKPDRAADVSMLMEEELSALLKTSRNV 200
lynx_Canada_gi|229893628|gb|FJ	STEDSGEDYSEGNASSNRGYSFGLKPDRAADVSMLMEEELSALLKTSRNV 200
Dog_Hungary_gi|34576246|emb|AJ	STEDSGEDYSEGNDSSNWGYSFGLKPDRAADVSMLMEEELSALLKTSRNV 200

dog_Thailand_gi|302128145|dbj|	STEDSGEDYSEGNASSNWGYSFGLKPDRAADVSMLMEEELSALLKTSRNV	200
fox_china_gb|HQ540293.1|_1694-	STEDSGEDYSEGNASSNWGYSFGLKPDRAADVSMLMEEELSALLKTSRNV	200
Dog_china_gb|HQ540292.1|_1694-	STEDSGEDYSEGNASSNWGYSFGLKPDRAADVSMLMEEELSALLKTSRNV	200
vaccineX_gi|158702386|gb|EU072	STEDSGEDYSEGNASSNWGYSFGLKPDRAADVSMLMEEELSALLRTSRNV	200
CDV_A75/17_gb|AF164967.1|_1801	STEDSGEDYSEGNASSNWGYSFGLKPDRAADVSMLMEEELSALLRTSRNV	200
Dog_USA_gb|EU716337.1|_1801-33	STEDSGEDYSEGNASSNWGYSFGLKPDRAADVSMLMEEELSALLKTSRTV	200
racoon_usa_gb|AY649446.1|_1801	STEDSGEDYSEGNASSNWGYSFGLKPDRAADVSMLMEEELSALLRTSRNV	200
Onderstepoort_gb|AF378705.1|_1	STEDSGEDYSEGNASSNWGYSFGLKPDRAADVSMLMEEELSALLRTSRNV	200
Duramine	STEDSGEDYSEGNASSNWGYSFGLKPDRAADVSMLMEEELSALLRTSKNV	200
Rockborn_gb|AF181446.1|	STEDSGEDYSEGNASSNWGYSFGLKPDRAADVSMLMEEELSALLRTSRNV	200
recombinant_Snyder_Hill_gb|GU1	STEDSGEDYSEGNASSNWGYSFGLKPDRAADVSMLMEEELSALLKTSRNV	200
Shuskiy_mink_gb|HM063009.1|_18	STEDSGEDYSEGNASSNWGYSFGLKPDRAADVSMLMEEELSALLKTSRNV	200
seal_Kazakhstan_gb|HM046486.1|	STEDSGEDYSEGNASSNWGYSFGLKPDRAADVSMLMEEELSALLKTSRNV	200
Monkey CYN07-dV (AB687720.2)	STEDSGEDYSEGNASSNWGYSFGLKPDRAADVSMLMEEELSALLKTSRNV	200

```
Dog (MN335914)                   GIQKRDGKTLQFPHNPEGKTGDPECGTIKKGTGERLASHGMGIVAGSTSG 250
Domestic dog (KJ415358)          GIQKRDGKTLQFPHNPEGKTGDPECGTIKKGTGERLASHGMGIVAGSTNG 250
Bat_earred_fox   (MN335913)      GIQKRDGKTLQFPHNPEGKTGDPECGTIKKGTGERLASHGMGIVAGSTSG 250
Bat eared fox (KJ415357)         GIQKRDGKTLQFPHNPEGKTGDPECGTIKKGTGERLASHGMGIVAGSTNG 250
African Lion (KJ415362)          GIQKRDGKTLQFPHNPEGKTGDPECGTIKKGTGERLASHGMGIVAGSTNG 250

Lion1 (MN335916)                 GIQKRDGKTLQFPHNPEGKTGDPECGTIKKGTGKRLASHGMGIVAGSTSG 250
Hyena1 (MN335918)                GIQKRDGKTLQFPHNPEGKTGDPECGTIKKGTGERLASHGMGIVAGSTSG 250
Hyena2 (MN335915)                -------------------------------------------------- 250
Spotted hyena (KU578258)         GIQKRDGKTLQFPHNPEGKTGDPECGTIKKGTGERLASHGMGIVAGSTNG 250
African_wild_dog_gi|169218409|   GIQKRDGKTLQFPHNPEGKTGDPECGTIKKGTGERLASHGMGIVAGS--- 250
dog_genbank_gi|4204842|gb|U537   GIQKRDGKTLQFPHNPEGKTGDPECGTIKKGTGERLASHGMGIVAGS--- 250
007japanease_dog_isolategi|787   GVQKRDGKTLQFPHNPEGKTGDPECGSIKKGTGEKSASHGMGIVAGSTSG 250
dog_Japan_gi|163838503|dbj|AB2   GIQKRDGKTLQFPHNPEGKTGDPECGSIKKGTGEKSASHGMGIVAGST-- 250
Marten_German_gi|34576254|emb|   GIQKRDGRTLQFPHNPEGKTGDPECGSIKKGTGERSASHGMGIVAGS--- 250
Iberian_lynxes_gi|299931630|gb   GIQKRDGKTLQFPHNPEGKTGDPGCGSIKKGTGERSASHGMEIVAGSTNG 250
German_dog2_gb|AY386315.1|       GIQKRDGKTLQFPHNPEGKTGDPECGSIKKGTGERSASHGMEIVAGSTNG 250
German_dog1_gi|34576244|emb|AJ   GIQKRDGKTLQFPHNPEGKTGDPECGSIKKGTGERSASHGMEIVAGS--- 250
German_red_fox_gi|341626873|gb   GIQKRDGKILQFPHNPEGKTGDPECGSIKKGTGERSASHGMEIVAGS--- 250
lynx_Canada_gi|229893628|gb|FJ   GIQKRDGKTLQFPHNPEGKTGDPECGSIKKGTGERSASHGMGIVAGS--- 250
Dog_Hungary_gi|34576246|emb|AJ   GIQKRDGKTLQFPHNPEGKTGDPECGSIKKGTGERSASHGTGIVAGS--- 250
```

```
dog_Thailand_gi|302128145|dbj|          GIKKRDGMTLQFPHNPEGKTEDPECGSIKKGTGERSASHGMGIVAGS--- 250
fox_china_gb|HQ540293.1|_1694-          GIKKRDGMTLQFPHNPEGKTEDPECGSIKKGTGERSASHGMGIVAGSTNG 250
Dog_china_gb|HQ540292.1|_1694-          GIKKRDGMTLQFPHNPEGKTEDPECGSIKKGTGERSASHGMGIAAGSTNG 250
vaccineX_gi|158702386|gb|EU072          GIQKRDGKTLQFPHNPEGKTGDPECGSIKKGTGERSASHGMGIVAGSTNG 250
CDV_A75/17_gb|AF164967.1|_1801          GIQKRDGKTLQFPRNPEGKTGDPECGSIKKGTGERSASHGMGIVAGSTNG 250
Dog_USA_gb|EU716337.1|_1801-33          GIQKRDGKTLQFPHNPEGKTGDPGCGSIKKGTGERSASHGMGIVAGSTNG 250
racoon_usa_gb|AY649446.1|_1801          GTQKRDGKTLQFPHNSESKTGDPECGSIKKGTGERSASHGTGIVAGSTNG 250
Hyena2                                  GIQKRDGKTLQFPHNPEGKTGDPECGTIKKGTER---------------- 250
Onderstepoort_gb|AF378705.1|_1          GIQKRDGKTLQFPHNPEGKTRDPECGSIKKGTEERSVSHGMGIVAGSTSG 250
Duramine                                GIQKRDGKTLQFPHNPRR-------------------------------- 250
Rockborn_gb|AF181446.1|                 GIQKRDGKTLQFPHNPEGKTRDPECGSIKKGTEERSVSHGMGIVAGSTSG 250
recombinant_Snyder_Hill_gb|GU1          GIQKRDGKTLQFPHNPEGKTRVPECGSIKKGTEERSVSHGMGIVAGSTSG 250
Shuskiy_mink_gb|HM063009.1|_18          GIQKRDGKTLQFPHNPEGKTRVPECGSIKKGTEERSVSQGMGIVAGSTSG 250
seal_Kazakhstan_gb|HM046486.1|          GIQKRDGKTLQFPHNPEGKTRVPECGSIKKGTEERSVSQGMGIVAGSTSG 250
Monkey CYN07-dV (AB687720.2)            GIKKRDGKTLQFPHNPEGKTEDPECGSIKKGTGERSASHGMGIVAGSTNG 250
```

Supplementary Figure 2

Alignment of CDV C amino acid sequences. Highlighted residues: yellow, synapomorphies shared by the Serengeti strains; red, consensus sequence; green, differences from the consensus.

```
Dog_USA_gb|EU716337.1|_1801-33      MSAKGWNASKPSERILLTLRRFKRSAASETKPATQARRMEPQACRKRRSL 50
racoon_usa_gb|AY649446.1|_1801      MSAKGWNASKPSERILLTLRRFKRSAASETKPATQARRMEPQACRKRRSL 50
CDV_A75/17_gb|AF164967.1|_1801      MSAKGWNASKPSERILLTLRRFKRSAASETKPATQARRMEPQACRKRRSL 50
vaccineX_gi|158702386|gb|EU072      MSVKGWNASKPSERILLTLRRFKRSAASETKPATQAKRMEPQACRKRRSL 50
Rockborn_gb|AF181446.1|_43-156      MSAKGWNASKPSERILLTLRRFKRSAASETKPATQAKRMEPQACRKRRSL 50
Onderstepoort_gb|AF378705.1|_1      MSAKGWNASKPSERILLTLRRFKRSAASETKPATQAKRMEPQACRKRRSL 50
Shuskiy_mink_gb|HM063009.1|_18      MSAKGWNASKPSERILLTLRRFKRSAASDTKPATQAKRMEPQACRKRRSL 50
seal_Kazakhstan_gb|HM046486.1|      MSAKGWNASKPSERILLTLRRFKRSAASDTKPATQAKRMEPQACRKRRSL 50
recombinant_Snyder_Hill_gb|GU1      MSAKGWNASKPSERILLTLRRFKRSAASETKPATQAKRMEPQACRKRRSL 50
007japanease_dog_isolategi|787      -------------------------------------------------- 50
Vaccine Duramine (MN400967)         MSARAG-CLKPSERILLTLRRFKRSAASETKPATQAKRMEPQACRKRRSL 50
dog_Japan_gi|163838503|dbj|AB2      -------------------------------------------------- 50
Iberian_lynxes_gi|299931630|gb      -------------------------------------------------- 50
German_dog1_gi|34576244|emb|AJ      -------------------------------------------------- 50
Marten_German_gi|34576254|emb|      -------------------------------------------------- 50
German_dog2_gb|AY386315.1|          MSAKGWNASKPSERILLTLRRFKRSAASETKPATQARRMEPQACRKRRSL 50
lynx_Canada_gi|229893628|gb|FJ      -------------------------------------------------- 50
fox_china_gb|HQ540293.1|_1694-      MSIKGWNASKPSERILLTLRRFKRSAVSEIKPATQAKRMEPQACRKKRSL 50
Dog_china_gb|HQ540292.1|_1694-      MSIKGWNASKPSERILLTLRRFKRSAVSEIKPATQAKRMEPQVCRKKRSL 50
dog_Thailand_gi|302128145|dbj|      -------------------------------------------------- 50
Dog_Hungary_gi|34576246|emb|AJ      -------------------------------------------------- 50
```

```
German_red_fox_gi|341626873|gb      -------------------------------------------------- 50

Dog (MN335914)                      -------------------------------------------------- 50

Lion 1 (MN335916)                   -------------------------------------------------- 50

Bat_eared_fox (MN335913)            -------------------------------------------------- 50

Hyena_2 (MN335915)                  --------KPSERILLTLRRFKRSAASETTPATQVKRMEPQACRKKRSL  50

Hyena1 (MN335918)                   -------------------------------------------------- 50

African_wild_dog_gi|169218409|      -------------------------------------------------- 50

Monkey CYN-dV (BAM15590.1)          MSTKGWNASKPSERILLTLRRFKRSAVSEIKPATQAKRMEPQACRKRRSL 50
```

Dog_USA_gb|EU716337.1|_1801-33	RISMNHTSQQKDQTMSAMYSKIIRDVERATLRLWRQSSPLKMTSNQDLEY	100
racoon_usa_gb|AY649446.1|_1801	RISMNHTSQQKDQTMSAMYSKIIREVERATLRLWRQSSPLKMTSNQDLEY	100
CDV_A75/17_gb|AF164967.1|_1801	RISMNHTSQQKDQTMSAMYSKIIRDVERATLRLWRQSSPLKMTSNQDLEY	100
vaccineX_gi|158702386|gb|EU072	RISMNHTSQQKDQTMSAMYSKIIRDVERATLRLWRQSSPLKMTSNQDLEY	100
Rockborn_gb|AF181446.1|_43-156	RISMNHTSQQKDQTMSAMYLKIIRDVENAILRLWRRSGPLERTSNQDLEY	100
Onderstepoort_gb|AF378705.1|_1	RISMNHTSQQKDQTMSAMYPKIIRDVENAILRLWRQSSPLKRTSNQDLEY	100
Shuskiy_mink_gb|HM063009.1|_18	RISMNHTSQQKDQTMSAMYSKIIRDVEKATLRLWKQSSSLKRTSNQDLEY	100
seal_Kazakhstan_gb|HM046486.1|	RISMNHTSQQKDQTMSAMYSKIIRDVEKATLRLWKQSSSLKRTSNQDLEY	100
recombinant_Snyder_Hill_gb|GU1	RISMNHTSQQKDQTMSAMYSKIIRDVEKATLRLWKQSSPLKRTSNQDLEY	100
007japanease_dog_isolategi|787	--	100
Vaccine Duramine (MN400967)	RISMNHTSQQKDQTMSAMYPKIIRDVENAILRLWRQSSPLKRTSNQDLEY	100
dog_Japan_gi|163838503|dbj|AB2	--	100
Iberian_lynxes_gi|299931630|gb	---QDLEY	100
German_dog1_gi|34576244|emb|AJ	--	100
Marten_German_gi|34576254|emb|	--	100
German_dog2_gb|AY386315.1|	RISMNHTSQQKDQTMSAMYSKIIREVERATLRLWRQSSPLKMSSNQDLEY	100
lynx_Canada_gi|229893628|gb|FJ	--	100
fox_china_gb|HQ540293.1|_1694-	RISMNHTSQQKDQTMSAMYSKIIRDVERATLRLWRQSSPLKMMSNQDLEY	100
Dog_china_gb|HQ540292.1|_1694-	RISMNHTSQQKDQTMSAMYSKIIRDVERATLRLWRQSSPPKTMSNQDLEY	100
dog_Thailand_gi|302128145|dbj|	--	100
Dog_Hungary_gi|34576246|emb|AJ	--	100

```
German_red_fox_gi|341626873|gb      -------------------------------------------------- 100

Dog (MN335914)                      --------------------------------------------------100

Lion 1 (MN335916)                   -------------------------------------------------- 100

Bat_eared_fox (MN335913)            -------------------------------------------------- 100

hyena_2 (MN335915)                  RISMNHTSQQKDQTMSAMYSRIIRDVERATLRLWRQSSPLKMTSNQDLEY 100

Hyena1 (MN335918)                   -------------------------------------------------- 100

African_wild_dog_gi|169218409|      -------------------------------------------------- 100

Monkey CYN-dV (BAM15590.1)          RISMNHTSQQKDQTISAMYSKIIRDVERATLRLWRQSSPLRMTSNQDLEY 100
```

Dog_USA_gb|EU716337.1|_1801-33	DVIMFMITAVKRLRESKMLAVSWYLQALSVIENSREEKEALMIALRILAK	150
racoon_usa_gb|AY649446.1|_1801	DVIMFMITAVKRLRESKMLTVSWYLQALSVIEDSREEREALMIALRILAK	150
CDV_A75/17_gb|AF164967.1|_1801	DVIMFMITAVKRLRESKMLTVSWYLQALSVIEDSREEKEALMIALRILAK	150
vaccineX_gi|158702386|gb|EU072	DVIMFMITAVKRLRESKMLTVSWYLQALSVIEDSREEKEALMIALRILAK	150
Rockborn_gb|AF181446.1|_43-156	DVIMFMITAVKRLRESKMLTVSWSLQALSVIEDSREEKEALMIALRILAK	150
Onderstepoort_gb|AF378705.1|_1	DVIMFMITAVKRLRESKMLTVSWYLQALSVIEDSREEKEALMTALRILAK	150
Shuskiy_mink_gb|HM063009.1|_18	DVIMFMITAVKRLRESKMLTVSWYLQALSVIEDSRAEKEALMIALRILAK	150
seal_Kazakhstan_gb|HM046486.1|	DVIMFMITAVKRLRESKMLTVSWYLQALSVIEDSRAEKEALMIALRILAK	150
recombinant_Snyder_Hill_gb|GU1	DVIMFMITAVKRLRESKMLTVSWYLQALSVIEDSRAEKEALMIALRILAK	150
007japanease_dog_isolategi|787	---MFMITAVKRLRESKMLTVSWYLQALSVIEDSREEKEALMIALRILAK	150
Vaccine Duramine (MN400967)	DVIMFMITAVKRLRESKMLTVSWYLQALSVIEDSREEKEALMIALRILAK	150
dog_Japan_gi|163838503|dbj|AB2	----------KRLRESKMLTVSWYLQALSVIEDSREEKEALMIALRILAK	150
Iberian_lynxes_gi|299931630|gb	DVIMFMITAVKRLRESKMLTVSWYLQALSVIEDSREEKEALMIALRILAK	150
German_dog1_gi|34576244|emb|AJ	----------KRLRESKMLTVSWYLQALSVIEDSREEKEALMIALRILAK	150
Marten_German_gi|34576254|emb|	----------KRLRESKMLTVSWYLQALSVIEDSREEKEALMIALRILAK	150
German_dog2_gb|AY386315.1|	DVIMFMITAVKRLRESKMLTVSWYLQALSVIEDSREEKEALMIALRILAK	150
lynx_Canada_gi|229893628|gb|FJ	----------KRLRESKMLTVSWYLQALSVIEDSREEKEALMIALRILAK	150
fox_china_gb|HQ540293.1|_1694-	DVIMFMITVVKRLRESKMLTVSWYLQALSVIEDSREEKEALMIALRILAK	150
Dog_china_gb|HQ540292.1|_1694-	DVIMFMITVVKRLRESKMLTVSWYLQALSVIEDSREEKEALMIALRILAK	150
dog_Thailand_gi|302128145|dbj|	----------KRLRESKMLTVSWYLQALSVIEDSREEKEALMIALRILAK	150
Dog_Hungary_gi|34576246|emb|AJ	----------KRLRESKMLTVSWYLQALSVIEDSREEKEALMIALRILAK	150

German_red_fox_gi|341626873|gb	------------KRLRVSKMLTVSWYLQALSVIEDSREEKEALMIALRILAK	150
Dog (MN335914)	--------------SSLSQMLTVSWYLQALSVIEDSREEKEALMIALRILAK	150
Lion 1 (MN335916)	--------------SYLSQMLTVSWYLQALSVIEDSREEKEALMIALRILAK	150
Bat_eared_fox (MN335913)	---------------YASQMLTVSWYLQALSVIEDSREEKEALMIALRILAK	150
hyena_2 (MN335915)	DVIMFMITAVKRLRESQMLTVSWYLQALSVIEDSREEKEALMIALRILAK	150
Hyena1 (MN335918)	--------------LRVSQMLTVSWYLQALSVIEDSREEKEALMIALRILAK	150
African_wild_dog_gi|169218409|	------------KRLRESQMLTVSWYLQALSVIEDSREEKEALMIALRILAK	150
Monkey CYN-dV (BAM15590.1)	DVIMFMITVVKRLRESKMLTVSWYLQALSVIEDSREEKEALMIALRILAK	150

Dog_USA_gb|EU716337.1|_1801-33	IIPREMLHLTGDILSALNQTEQLM	174
racoon_usa_gb|AY649446.1|_1801	IIPREMLHLTGDILSALNQTGQLM	174
CDV_A75/17_gb|AF164967.1|_1801	IIPREMLHLTGDILSALNQTEQLM	174
vaccineX_gi|158702386|gb|EU072	IIPREMLHLTGDILSALNQTEQLM	174
Rockborn_gb|AF181446.1|_43-156	IIPKEMLHLTGDILSALNRTEQLM	174
Onderstepoort_gb|AF378705.1|_1	IIPKEMLHLTGDILSALNRTEQLM	174
Shuskiy_mink_gb|HM063009.1|_18	IIPKEMLHLTGDILSALNQTGQLM	174
seal_Kazakhstan_gb|HM046486.1|	IIPKEMLHLTGDILSALNQTGQLM	174
recombinant_Snyder_Hill_gb|GU1	IIPKEMLHLTGDILSALNQTGQLM	174
007japanease_dog_isolategi|787	IIPKEMLHLTGDILSALNQTEQPM	174
Vaccine_Duramine (MN400967)	IIPKEMLHLTGDILSALNRTEQLM	174
dog_Japan_gi|163838503|dbj|AB2	IIPKEMLHLTGDILSALNQTEQPM	174
Iberian_lynxes_gi|299931630|gb	IIPREMLHLTGDILSALNQTEQLM	174
German_dog1_gi|34576244|emb|AJ	IIPREMLHLTGDILSALNQTEQLM	174
Marten_German_gi|34576254|emb|	IIPREMLHLTGDILSALNQTEQLT	174
German_dog2_gb|AY386315.1|	IIPREMLHLTGDILSALNQTEQLM	174
lynx_Canada_gi|229893628|gb|FJ	IIPREMLHLTGDILSALNQTEQLM	174
fox_china_gb|HQ540293.1|_1694-	IIPREMLHLTGDILSALNQTERLM	174
Dog_china_gb|HQ540292.1|_1694-	IIPREMLHLTGDILSALNQTERLM	174
dog_Thailand_gi|302128145|dbj|	IIPREMLHLTGDILSALNQTERLM	174
Dog_Hungary_gi|34576246|emb|AJ	IIPREMIHLTGDILSALNQTEQLM	174

German_red_fox_gi|341626873|gb	IIPREMLHLTGDILSALNQTEQLM	174
Dog (MN335914)	IIPREMLHLTGDILSVLNQTEQLM	174
Lion 1 (MN335916)	IIPREMLHLTGDILSVLNQTEQLM	174
Bat_eared_fox (MN335913)	IIPREMLHLTGDILSVLNQTEQLM	174
hyena_2 (MN335915)	IIPREMLHLTGDILSVLNQTEQLM	174
Hyena1 (MN335918)	IIPREMLHLTGDILSVSNQTEQLM	174
African_wild_dog_gi|169218409|	IIPREMLHLTGDILSVLNQTEQLM	174
Monkey CYN-dV (BAM15590.1)	IIPREMLHLTGDILSALNQTERLM	174

Supplementary Figure 3   H Sequence Alignment

Alignment of CDV H amino acid sequences. Highlighted residues: yellow, synapomorphies shared by the Serengeti strains; red, consensus sequence; magenta, differences from the consensus; turquoise specific to monkey virus. Substitutions in PDV have not been highlighted.Boxes indicate: red, residues critical for SLAM binding; blue, residue critical for Nectin-4 binding; green, site suggested by McCarthy et al.[56] to be associated with spread to non-canine host.

```
Rockborn-Candurgb|GU266280.1|_      MLSYQDKVGAFYKDNARANSSKLSLVTEEQGGRRPPYLLFVLLILLVGIM 50
Lesser_panda_gi|5870827|gb|AAD      MLSYQDKVGAFYKDNARANSSKLSLVTEEQGGRRPPYLLFVLLILLVGIM 50
Vanguard_vaccine_gi|239949423|      MLSYQDKVGAFYKDNARANSSKLSLVTEEQGGRRPPYLLFVLLILLVGIM 50
Vacc-P_gi|224579344|gb|FJ70523      MLSYQDKVGAFYKDNARANSSKLSLVTEEQGGRRPPYLLFVLLILLVGIM 50
Dog_argentina_gi|134801370|emb      -------------------------------------------------- 50
German_dog_gi|693948|emb|X8500      MLSYQDKVGAFYKDNARANSSKLSLVTEEQGGRRPPYLLFVLLILLVGIM 50

African Lion (JN812975)             MLSHQDKVGAFYKDNARANSSKLSLMTEERGDRRPPYLLFVLLILLVGIL 50
Lion1 (MN335911)                    --------------------------------------------------
Hyena2 (MN335910)                   MLSHQDKVGAFYKDNARANSSKLSLMTEERGDRRPPYLLFVLLILLVGIL 50
Spotted Hyena (KJ415364)            -------------------------------------------------- 50
Bat_Eared_Fox (MN335908)            -------------DNARANSSKLSLMTEERGDRRPPYLLFVLLILLVGIL 50

Bat Eared fox (KC916716)            MLSHQDKVGAFYKDNARANSSKLSLMTEERGDRRPPYLLFVLLILLVGIL 50
DOG_H.2_1 (MN335909)                -------------------------------------------------- 50

Domestic dog (JN812976)             MLSHQDKVGAFYKDNARANSSKLSLMTEERGDRRPPYLLFVLLILLVGIL 50
Hyena1    (MN335912)                -------------------------------------------------- 50
Monkey (CYN07-dV(AB687720.2)        MLSYQDKVGAFYKDNARANSSKLFLVTEEQGGRRPPYLLFVLLILLIGIL 50
A75/17 (AF164967.1)                 MLSYQDKVSAFYKDNARANSSKLSLVTEEQGGRRPPYLLFVLLILLVGIM 50
Dog_ChinaB_gi|212656655|gb|ACJ      MLSYQDKVGAFYKDNARANSSKLSLVTEEQGGRRPPYLLFVLLILLIGIL 50
German_ferret_gi|693949|emb|X8      MLSYQDKVGAFYKDNARANSSKLSLVTEEQGGRRPPYLLFVLLILLVGIM 50
South_African_dog_gi|239949465      MLSYQDKVGAFYKDNARANSSKLSLVTEEQGIRRPPYLLFVLLILLVGVL 50
Dog_South_Africa_c_gi|23994946      MLSYQDKVGAFYKDNARANSSKLSLVTEEQGIRRPPYLLFVLLILLVGVL 50
Japanease_dogstrain_007Lmgi|78      MLSYQDKVGAFYKDNARANSSKLSLVTEEQGGRRPPYLLFVLLILLVGIL 50
```

Onderstepoort_gb|AF378705.1|	MLSYQDKVGAFYKDNARANSTKLSLVTEEHGGRRPPYLLFVLLILLVGIL 50
Convac_vaccine_gi|517242|emb|Z	MLSYQDKAGAFYKDNARANSTKLSLVTEEHGGRRPPYLLFVLLVLLVGIL 50
Phoca/Caspian/2007/HM046486	MLSYQDKVGAFYKDNARANSSKLSPVTEEHGGRRPPYLLFVLLILLVGIL 50
PDV/ USA/ 2006/HQ007902.1	MFSHQDKVGAFYKNNARANSSKLSLVTDEVEERRSPWFLSILLILLVGIL 50

Rockborn-Candurgb|GU266280.1|_	ALLAITGVRFHQVSTSNMEFSRLLKEDMEKSEAVHHQVIDVLTPLFKIIG	100
Lesser_panda_gi|5870827|gb|AAD	ALLAITGVRFHQVSTSNMEFSRLLKENMEKSEAVHHQVIDVLTPLFKIIG	100
Vanguard_vaccine_gi|239949423|	ALLAITGVRFHQVSTSNMEFSRLLKEDMEKSEAVHHQVIDVLTPLFKIIG	100
Vacc-P_gi|224579344|gb|FJ70523	ALLAITGVRFHQVSTSNMEFSRLLKEDMEKSEAVHHQVIDVLTPLFKIIG	100
Dog_argentina_gi|134801370|emb	--	100
German_dog_gi|693948|emb|X8500	TLLAITGVRFHQVSTSNMEFSRLLKEDMEKSEAVHHQVIDVLTPLFKIIG	100
African Lion (JN812975)	ALLAITGVRFHQVSTSNMEFSRLLKEDMEKSEAVHHQVIDVLTPLFKIIG	100
Lion1 (MN335911)	--	100
hyena2 (MN335910)	ALLAITGVRFHQVSTSNMEFSRLLKEDMEKSEAVHHQVIDVLTPLFKIIG	100
Spotted Hyena (KJ415364)	--	100
Bat_Eared_Fox (MN335908)	ALLAITGVRFHQVSTSNMEFSRLLKEDMEKSEAVHHQVIDVLTPLFKIIG	100
Bat Eared fox (KC916716)	ALLAITGVRFHQVSTSNMEFSRLLKEDMEKSEAVHHQVIDVLTPLFKIIG	100
DOG_H.2_1 (MN335909)	--	100
Domestic dog (JN812976)	ALLAITGVRFHQVSTSNMEFSRLLKEDMEKSEAVHHQVIDVLTPLFKIIG	100
hyena1 (MN335912)	--	100
Monkey(CYN07-dV(AB687720.2)	ALLAITGVRFHQVSTSNMEFSRLLKEDMEKSEAVHHQVIDVLTPLFKIIG	100
A75/17 (AF164967.1)	ALLAITGVRFHQVSTSNMEFSRLLKEDMEKSEAVHHQVIDVLTPLFKIIG	100
Dog_ChinaB_gi|212656655|gb|ACJ	ALLAITGVRFHQVSTSNMEFSRLLKEDMEKSEAVHHQVIDVLTPLFKIIG	100
German_ferret_gi|693949|emb|X8	ALLAITGVRFHQVSTSNMEFSRLLKEDMDRSEAVHHQVIDVLTPLFKIIG	100
south_african_dog_gi|239949465	ALLAITGVRFHQVSTSNMEFSRLLKEDMEKSEAVHHQVIDVLTPLFKIIG	100
Dog_South_Africa_c_gi|23994946	ALLAITGVRFHQVSTSNMEFSRLLKEDMEKSEAVHHQVIDVLTPLFKIIG	100
Japanease_dogstrain_007Lmgi|78	ALLAIAGVRFRQVSTSNVEFGRLLKDDLEKSEAVHHQVMDVLTPLFKIIG	100

Onderstepoort_gb|AF378705.1|	ALLAITGVRFHQVSTSNMEFSRLLKEDMEKSEAVHHQVIDVLTPLFKIIG	100
Convac_vaccine_gi|517242|emb|Z	ALLAITGVRFHQVSTSNMEFSRLLKEDMEKSEAVHHQVIDVLTPLFKIIG	100
Phoca/Caspian/2007/HM046486	ALLAITGVRFHQVSTSNMEFSRLLKEDMEKSEAVHHQVIDVLTPLFKIIG	100
PDV/ USA/ 2006/HQ007902.1	ILLAITGIRFHQVVKSNLEFNKLLIEDMEKTEAVHHQVKDVLTPLFKIIG	100

Rockborn-Candurgb|GU266280.1|_	DEIGLRLPQKLNEIKQFILQKTNFFNPNREFDFRDLHWCINPPSKIKVNL	150
lesser_panda_gi|5870827|gb|AAD	DEIGSRLPQKLNEIKQFILQKTNFFNPNREFDFRDLHWCINPPSKIKVNL	150
Vanguard_vaccine_gi|239949423|	DEIGLRLPQKLNEIKQFILQKTNFFNPNREFDFRDLHWCINPPSKIKVNL	150
Vacc-P_gi|224579344|gb|FJ70523	DEIGLRLPQKLNEIKQFILQKTNFFNPNREFDFRDLHWCINPPSKIKVNL	150
Dog_argentina_gi|134801370|emb	--	150
German_dog_gi|693948|emb|X8500	DEVGLRLPQKLNEIKQFILQKTNFFNPNREFDFRDLHWCINPPSKIKVNF	150
African Lion (JN812975)	DEIGLRLPQKLNEIKQFILQKTNFFNPNREFDFRDLHWCINPPSKIKVNF	150
Lion 1 (MN335911)	--	150
Hyena 2 (MN335910)	DEIGLRLPQKLNEIKQFILQKTNFFNPNREFDFRDLHWCINPPSKIKVNF	150
Spotted Hyena (KJ415364)	--	150
Bat_Eared_Fox (MN335908)	DEIGLRLPQKLNEIKQFILQKTNFFNPNREFDFRDLHWCINPPSKIKVNF	150
Bat Eared fox (KC916716)	DEIGLRLPQKLNEIKQFILQKTNFFNPNREFDFRDLHWCINPPSKIKVNF	150
DOG_H.2_1 (MN335909)	--	150
Domestic dog (JN812976)	DEIGLRLPQKLNEIKQFILQKTNFFNPNREFDFRDLHWCINPPSKIKVNF	150
Hyena1 (MN335912)	--	150
Monkey (CYN07-dV(AB687720.2)	DEIGLRLPQKLNEIKQFILQKTNFFNPNREFDFRDLHWCINPPSKIKVNF	150
A75/17 (AF164967.1)	DEIGLRLPQKLNEIKQFILQKTNFFNPNREFDFRDLHWCINPPSKIKVNF	150
Dog_ChinaB_gi|212656655|gb|ACJ	DEIGLRLPQKLNEIKQFILQKTNFFNPNREFDFRDLHWCINPPSKIKVNF	150
German_ferret_gi|693949|emb|X8	DEIGLRLPQKLNEIKQFILQKTNFFNPNREFDFRDLHWCINPPSKIKVNF	150
south_african_dog_gi|239949465	DEIGLRLPQKLNEIKQFILQKTNFFNPSREFDFRDLHWCINPPSKIKVNF	150
Dog_South_Africa_c_gi|23994946	DEIGLRLPQKLNEIKQFILQKTNFFNPSREFDFRDLHWCINPPSKIKVNF	150

```
Japanease_dogstrain_007Lmgi|78      DEIGLRLPQKLNEIKQFILQKTNFFNPNREFDFRDLHWCINPPSKIKVNF 150
Onderstepoort_gb|AF378705.1|        DEIGLRLPQKLNEIKQFILQKTNFFNPNREFDFRDLHWCINPPSKVKVNF 150
Convac_vaccine_gi|517242|emb|Z      DEIGSRLPQKLNEIKQFILQKTNFFNPNREFDFRDLHWCINPPSKVKVNF 150
Phoca/Caspian/2007/HM046486         DEIGLRLPQKLNEIKQFILQKTNFFNPNREFDFRDLHWCINPPSKVKVNF 150
PDV/ USA/ 2006/HQ007902.1           DEVGLRLPQKLNEIKQFIVQKTNFFNPNREFDFRELHWCINPPSKVKVNF 150
Rockborn-Candurgb|GU266280.1|_      TNYCDTIGLRKSIASAANPILLSALSRGRGDIFPPYRCSGATTSVGRFFP 200
lesser_panda_gi|5870827|gb|AAD      TNYCDTIGLRKSIASAANPILLSALSRGRGDIFPPYRCSGATTSVGRFFP 200
Vanguard_vaccine_gi|239949423|      TNYCDTIGLRKSIASAANPILLSALSRGRGDIFPPYRCSGATTSVGRFFP 200
Vacc-P_gi|224579344|gb|FJ70523      TNYCDTIGLRKSIASAANPILLSALSRGRGDIFPPYRCSGATTSVGRFFP 200
Dog_argentina_gi|134801370|emb      -------------------------------------------------- 200
German_dog_gi|693948|emb|X8500      TNYCDTIGIKKSIASAANPILLSALSGGRGDIFPPYRCSGATTSVGRVFP 200
African Lion (JN812975)             TNYCDTIGIKKSIASAANPILLSALSGGRGDIFPPYRCSGATTSVGRVFP 200
Lion 1 (MN335911)                   -------------------------------------------------- 200
hyena2 (MN335910)                   TNYCDTIGIKKSIASAANPILLSALSGGRGDIFPPYRCSGATTSVGRVFP 200
Spotted Hyena (KJ415364)            -------------------------------------------------- 200
Bat_Eared_Fox (MN335908)            TNYCDTIGIRKSIASAANPILLSALSGDRGDIFPPYRCSGATTSVGRVFP 200
Bat Eared Fox (KC916716)            TNYCDTIGIRKSIASAANPILLSALSGDRGDIFPPYRCSGATTSVGRVFP 200
DOG_H.2_1 (MN335909)                -------------------------------------------------- 200
Domestic dog (JN812976)             TNYCDTIGIRKSIASAANPILLSALSGDRGDIFPPYRCSGATTSVGRVFP 200
hyena1 (MN335912)                   -------------------------------------------------- 200
Monkey(CYN07-dV(AB687720.2)         TNYCDTVGVKKSIASAANPIILSALSGARGDIFPPYRCSGATTSVGRVFP 200
A75/17 (AF164967.1)                 TNYCDTIGIRKSIASAANPILLSALSGGRGDIFPPYRCSGATTSVGRVFP 200
```

```
Dog_ChinaB_gi|212656655|gb|ACJ      TNYCDTVGVKKSIASAANPIILSALSGARGDIFPQYRCSGATTSVGRVFP 200
German_ferret_gi|693949|emb|X8      TNYCDTIGIRKSIASAANPILLSALSEGRGDIFPPYRCSGATTSVGRVFP 200
south_African_dog_gi|239949465      TSYCDTIGIKKSIASAAKPILLSALSGGRGDIFPPYRCSGATTSMGRVFP 200
Dog_South_Africa_c_gi|23994946      TSYCDTVGIRKSIALAAKPILLSALSGGKGDIFPPYRCSGATTSIGRVFP 200
Japanease_dogstrain_007Lmgi|78      TNYCDAIGVRKSIASAANPILLSALSGGRGDIFPPYRCSGATTSVGRVFP 200
Onderstepoort_gb|AF378705.1|        TNYCESIGIRKAIASAANPILLSALSGGRSDIFPPHRCSGATTSVGKVFP 200
Convac_vaccine_gi|517242|emb|Z      TNYCESIGIRKAIASAANPILLSALPGGRSDIFPPHRCSGATTSVGKVFP 200
Phoca/Caspian/2007/HM046486         TNYCETIGIRKSIASAANPILLSALSGGRSDIFPPYRCRGATTSVGKVFP 200
PDV/ USA/ 2006/HQ007902.1           TQYCEITEFKEAIRSVANSILLLTLYRGRDDIFPPYKCRGATTSMGNVFS 200
```

Rockborn-Candurgb|GU266280.1|_	LSVSLSMSLISRTSEIINMLTAISDGVYGKTYLLVPDYIEGEFDTQKIRV	250
lesser_panda_gi|5870827|gb|AAD	LSVSLSMSLISRTSEIINMLTAISDGVYGKTYLLVPDYIEGEFDTQKIRV	250
Vanguard_vaccine_gi|239949423|	LSVSLSMSLISRTSEIINMLTAISDGVYGKTYLLVPDYIEGEFDTQKIRV	250
Vacc-P_gi|224579344|gb|FJ70523	LSVSLSMSLISRTSEIINMLTAISDGVYGKTYLLVPDYIEGEFDTQKIRV	250
Dog_argentina_gi|134801370|emb	--	250
German_dog_gi|693948|emb|X8500	LSVSLSMSLISKTSEITSMLTAISDGVYGKTYLLVPDYIEGEFDTQKIRV	250
African Lion (JN812975)	LSVSLSMSLISRTAEIINMLTAISDGVYGKTYLLVPDYIEGEFDTQKIRV	250
Lion 1 (MN335911)	--	250
Hyena 2 (MN335910)	LSVSLSMSLISRTAEIINMLTAISDGVYGKTYLLVPDYIEGEFDTQKIRV	250
Spotted Hyena (KJ415364)	--	250
Bat_Eared_Fox (MN335908)	LSVSLSMSLISRTAEIINMLTAISDGVYGKTYLLVPDYIEGEFDTQKIRV	250
Bat Eared Fox (KC916716)	LSVSLSMSLISRTAEIINMLTAISDGVYGKTYLLVPDYIEGEFDTQKIRV	250
DOG_H.2_1 (MN335909)	--	250
Domestic dog (JN812976)	LSVSLSMSLISRTAEIINMLTAISDGVYGKTYLLVPDYIEGEFDTQKIRV	250
Hyena1 (MN335912)	--	250
Monkey(CYN07-dV(AB687720.2)	LSVSLSMSLISRTSEIINMLTAISDGVYGKTYLLVPDYIEGEFDSQKIRV	250
A75/17 (AF164967.1)	LSVSLSMSLISRTSEIINMLTAISDGVYGKTYLLVPDYIEGGFDTQKIRV	250
Dog_ChinaB_gi|212656655|gb|ACJ	LSVSLSMSLISRTSEIINMLTAISDGVYGKTYLLVPDYIEGEFDSQKIRV	250
German_ferret_gi|693949|emb|X8	LSVSLSMSLISRTSEIINMLTAISDGVYGKTYLLVPDYIEGEFDTQKIRV	250
south_african_dog_gi|239949465	LSVSLSMSLISRTSEIINMLTAISDGVYGKTYLLVPDYIEGEFDTQKIRV	250
Dog_South_Africa_c_gi|23994946	LSVSLSMSLISRTSEIINMLTAVSDGVYGKTYLLVPDYIEGEFDTQKIRV	250
Japanease_dogstrain_007Lmgi|78	LSVSLSMSLISKTSEIISMLTAISDGVYGKTYLLVPDYIEREFDTQKIRV	250

```
Onderstepoort_gb|AF378705.1|        LSVSLSMSLISRTSEIINMLTAISDGVYGKTYLLVPDDIEREFDTQEIRV 250

Convac_vaccine_gi|517242|emb|Z      LSVSLSMSLISRTSEIINMLTAISDGVYGKTYLLAPDDIEREFDTQEIRV 250

Phoca/Caspian/2007/HM046486         LSVSLSMSLISRTSEIINMLTATSDGVYGKTYLLVPDDIEREFDTQEIRV 250

PDV/ USA/ 2006/HQ007902.1           LAVSLSMSLISKPSEVITMLTAISEGIYGKTYLLVTDDTEENFETPEIRV 250
```

Rockborn-Candurgb|GU266280.1|_	FEIGFIKRWLNDMPLLQTTNYMVLPENSKAKVCTIAVGELTLASLCVDES	300
lesser_panda_gi|5870827|gb|AAD	FEIGFIKRWLNDMPSLQTTNYMVLPENSKAKVCTIAVGELTLASLCVDES	300
Vanguard_vaccine_gi|239949423|	FEIGFIKRWLNDMPLLQTTNYMVLPENSKAKVCTIAVGELTLASLCVDES	300
Vacc-P_gi|224579344|gb|FJ70523	FEIGFIKRWLNDMPLLQTTNYMVLPENSKAKVCTIAVGELTLASLCVDES	300
Dog_argentina_gi|134801370|emb	--	300
German_dog_gi|693948|emb|X8500	FEIGFIKRWLNNMPLLQTTNYMVLPENSKAKVCTIAVGELTLASLCVDES	300
African Lion (JN812975)	FEIGFIKRWLNNMPLLQTTNYMVLPENSKAKVCTIAVGELTLASLCVDES	300
Lion 1 (MN335911)	--	300
Hyena 2 (MN335910)	FEIGFIKRWLNNMPLLQTTNYMVLPENSKAKVCTIAVGELTLASLCVDES	300
Spotted Hyena (KJ415364)	--	300
Bat_Eared_Fox (MN335908)	FEIGFIKRWLNNMPLLQTPNYMVLPENSKAKVCTIAVGELTLAFLCVDES	300
Bat Eared Fox (KC916716)	FEIGFIKRWLNNMPLLQTTNYMVLPENSKAKVCTIAVGELTLASLCVDKS	300
DOG_H.2_1 (MN335909)	--	300
Domestic dog (JN812976)	FEIGFIKRWLNNMPLLQTTNYMVLPENSKAKVCTIAVGELTLASLCVDES	300
hyena1 (MN335912)	--	300
Monkey(CYN07-dV(AB687720.2)	FEIGFIKRWLNDMPLLQTTNYMVLPVTSKAKVCTIAVGELTLASLCVDES	300
A75/17 (AF164967.1)	FEIGFIKRWLNDMPLLQTTNYMVLPENSKAKVCTIAVGELTLASLCVDES	300
Dog_ChinaB_gi|212656655|gb|ACJ	FEIGFIKRWLNDMPLLQTTNYMVLPETSKAKVCTIAVGELTLASLCVDES	300
German_ferret_gi|693949|emb|X8	FEIGFIKRWLNDMPLLQTTNYMVLPENSKAKVCTIAVGELTLASLCVDES	300
south_african_dog_gi|239949465	FEIGFIKRWLNDMPLLQTTNYMVLPENSKAKVCTIAVDWVTLASLCVDDS	300
Dog_South_Africa_c_gi|23994946	FEIGFIKRWLNDMPLLQTTNYMVLPENSKAKVCTIAVGELTLASLCVGES	300
Japanease_dogstrain_007Lmgi|78	FEIGFIKRWLNDMPLLQTTNYMVLPENSKAKVCTIAVGELTLASLCVDES	300

Onderstepoort_gb|AF378705.1|	FEIGFIKRWLNDMPLLQTTNYMVLPENSKAKVCTIAVGELTLASLCVEES	300
Convac_vaccine_gi|517242|emb|Z	FEIGFIKRWLNDMPSLQTTNYMVLPENSKAKVCTIAVGELTLASLCVEES	300
Phoca/Caspian/2007/HM046486	FEIGFIKRWLNDMPLLQTTNYMVLPENSKAKVCTIAVGELTLASLCVEES	300
PDV/ USA/ 2006/HQ007902.1	FEIGFINRWLGDMPLFQTTNYRIISDNSNTKICTIAVGELALASLCTKES	300

```
Rockborn-Candurgb|GU266280.1|_      TVLLYHDSNGSQDGILVVTLGIFGATPMDQVEEVIPVAHPSVEKIHITNH 350
lesser_panda_gi|5870827|gb|AAD      TVLLYHDSNGSQDGILVVTLGIFGATPMDQVEEVIPVAHPSVEKIHITNH 350
Vanguard_vaccine_gi|239949423|      TVLLYHDSNGSQDGILVVTLGIFGATPMDQVEEVIPVAHPSVEKIHITNH 350
Vacc-P_gi|224579344|gb|FJ70523      TVLLYHDSNGSQDGILVVTLGIFGATPMDQVEEVILVAHPSVEKIHITNH 350
Dog_argentina_gi|134801370|emb      ----YHDGNGSQDGILVVTLGIFGATPMDQVEEVIPVAHPSVEKIHITNH 350
German_dog_gi|693948|emb|X8500      TVLLYHDSNGSQDGILVVTLGIFGATPMDQVEEVIPVAHPSVEKIHITNH 350
African Lion (JN812975)             TVLLSHDSNGSQDGILVVTLGIFGATLMDQVEEVIPVAHPSVEKIHITNH 350
Lion 1 (MN335911)                   -----HDSDGSQDGILVVTLGIFGATLMDQVEEVIPVAHPSVEKIHITNH 350
Hyena 2 (MN335910)                  TVLLSH SNGSQDGILVVTLGIFGATLMDQVEEVIPVAHPSVEKIHITNH 350
Spotted Hyena (KJ415364)            -------------------------------------------------- 350
Bat_Eared_Fox (MN335908)            TVLLLLMKHGSQDGILVVTLGIFGATLMDQVEEVIPVAHPSVEKIHITNH 350
Bat Eared Fox (KC916716)            TVLLSHDSNGSQDGILVVTLGIFGATLMDQVEEVIPVAHPSVEKIHITNH 350
DOG_H.2_1 (MN335909)                --TMHLMKHGSQDGILVVTLGIFGATLMDQVEEVIPVAHPSVEKIHITNH 350
Domestic dog (JN812976)             TVLLSHDSNGSQDGILVVTLGIFGATLMDQVEEVIPVAHPSVEKIHITNH 350
Hyena1 (MN335912)                   --------DGSPDGILVLTLGIFGATLMDQVEEVIPVAHPSVEKIHITNH 350
Monkey(CYN07-dV(AB687720.2)         TVLLYHDSNGSQNGILVVTLGIFGATPMDQVEEVIPVAHPSVERIHITNH 350
A75/17  (AF164967.1)                TVLLYHDSDGSQDGILVVTLGIFGATPMDQVEEVIPVAHPSVEKIHITNH 350
Dog_ChinaB_gi|212656655|gb|ACJ      TVLLYHDSNGSQDGILVVTLGIFGATPMDQVEEVIPIAHPSVERIHITNH 350
German_ferret_gi|693949|emb|X8      TVLLYHDSNGSQDGILVVTLGIFGATPMDQVEEVVPVAHSSVEKIHITNH 350
south_african_dog_gi|239949465      TVLLYHDSNGSQGSILVVTLGIFGATPMDQVEEVIPIAHPSVEKIHITNH 350
Dog_South_Africa_c_gi|23994946      TVLLYHDSNGSQDGILVVTLGIFGATPMDQVEEVIPVAHPSVEKIHITNH 350
Japanease_dogstrain_007Lmgi|78      TVLLYHDSNGSQDSILVVTLGIFGATPMNQVEEVIPVAHPSVERIHITNH 350
```

Onderstepoort_gb|AF378705.1|	TVLLYHDSSGSQDGILVVTLGIFWATPMDHIEEVIPVAHPSMEKIHITNH	350
Convac_vaccine_gi|517242|emb|Z	TVLLYHDSSGSQDGILVVTLGIFGTTPMDHIEEVIPVAHPSMEKIHITNH	350
Phoca/Caspian/2007/HM046486	TVLLYHDSRGSQDGILVVTLGIFGATPMDHIEEVIPVAHPSMEKIHITNH	350
PDV/ USA/ 2006/HQ007902.1	TILLNLGDEESQNSVLVVILGLFGATHMDQLEEVIPVAHPSIEKIHITNH	350

Rockborn-Candurgb|GU266280.1|_	RGFIKDSIATWMVPALVSEKQEEQKNCLESACQRKSYPMCNQTSWEPFGG	400
lesser_panda_gi|5870827|gb|AAD	RGFIKDSIATWMVPALVSEKQEEQKNCLESACQRKSYPMCNQTSWEPFGG	400
Vanguard_vaccine_gi|239949423|	RGFIKDSIATWMVPALVSEKQEEQKNCLESACQRKSYPMCNQTSWEPFGG	400
Vacc-P_gi|224579344|gb|FJ70523	RGFIKDSIATWMVPALVSEKQEEQKNCLESACQRKSYPMCNQTSWEPFGG	400
Dog_argentina_gi|134801370|emb	RGFIKDSKAIWMVPALVSEKQEEQKNCLESACQRKTYPMCNQTSWEPFGG	400
German_dog_gi|693948|emb|X8500	RGFIKDSIATWMVPALVSEKQEEQKNCLESACQRKTYPMCNQTSWEPFGG	400
African Lion (JN812975)	RGFIKDSIATWMVPALASEKQEEQQNCLESACQRKSYPMCNQTSWEPFGG	400
Lion 1 (MN335911)	RGFIKDSIATWMVPALASEKQEEQQNCLESACQRKSYPMCNQTSWEPFGG	400
Hyena 2 (MN335910)	RGFIKDSIATWMVPALASEKQEEQQNCLESACQRKSYPMCNQTSWEPFGG	400
Spotted Hyena (KJ415364)	--	400
Bat_Eared_Fox (MN335908)	RGFIKDSIATWMVPALASEKQEEQQNCLESACQRKSYPMCNQTSWEPFGG	400
Bat Eared Fox (KC916716)	RGFIKDSIATWMVPALASEKQEEQQNCLESACQRKSYPMCNQTSWEPFGG	400
DOG_H.2_1 (MN335909)	RGFIKDSIATWMVPALASEKQEEQQNCLESACQRKSYPMCNQTSWEPFGG	400
Domestic dog (JN812976)	RGFIKDSIATWMVPALASEKQEEQQNCLESACQRKSYPMCNQTSWEPFGG	400
Hyena 1 (MN335912)	RGFIKDSISTWMVPALASEKQEEQQNCLESACQRKSYPMCNQTSWEPFGG	400
Monkey(CYN07-dV(AB687720.2)	RGFIKDSIVTWMVPALVSEKQEEQKNCLESACQRKSYPMCNRTSWEPFGG	400
A75/17 (AF164967.1)	RGFIKDSIATWMVPALVSEKQEEQKNCLESACQRKSYPMCNQTSWEPFGG	400
Dog_ChinaB_gi|212656655|gb|ACJ	RGFIKDSIVTWMVPVLVSEKQEEQKNCLESACHRKSYPMCNQTSWEPFGG	400
German_ferret_gi|693949|emb|X8	RGFIKDSIATWMVPALVSEKQEEQKNCLESACQRKSYPMCNQTSWEPFGG	400
south_african_dog_gi|239949465	RGFIKDSIATWMVPALVSERQEEQKICLESACQRKSYPMCNQTSWEPFGG	400
Dog_South_Africa_c_gi|23994946	RGFIKDSIATWMVPALVSERQEEQKICLESACQRKSYPMCNQTSWEPFGG	400

```
Japanease_dogstrain_007Lmgi|78      RGFIKDSVATWMVPALVSEQQEGQKNCLESACQRKSYPMCNQTSWEPFGG 400
Onderstepoort_gb|AF378705.1|        RGFIKDSIATWMVPALASEKQEEQKGCLESACQRKTYPMCNQTSWEPFGG 400
Convac_vaccine_gi|517242|emb|Z      RGFIKDSIATWMVPALASEKQEEQKGCLESACQRKPYPMCNQTSWEPFGG 400
Phoca/Caspian/2007/HM046486         RGFIKDSIATWMVPALASEKQEEQKGRLESACQRKTYPMCNQTSWEPFGG 400
PDV/ USA/ 2006/HQ007902.1           RGFIKDSVATWMVPALALSEQGEQINCLRSACKRRTYPMCNQTSWEPFDD 400
```

Rockborn-Candurgb|GU266280.1|_	GQLPSYGRLTLPLDPSIDLQLNISFTYGPVILNGDGMDYYESPLLDSGWL	450
lesser_panda_gi|5870827|gb|AAD	GQLPSYGRLTLPLDPSIDLQLNISFTYGPVILNGDGMDYYESPLLDSGWL	450
Vanguard_vaccine_gi|239949423|	GQLPSYGRLTLPLDPSIDLQLNISFTYGPVILNGDGMDYYESPLLDSGWL	450
Vacc-P_gi|224579344|gb|FJ70523	GQLPSYGRLTLPLDPSIDLQLNISFTYGPVILNGDGMDYYESPLLDSGWL	450
Dog_argentina_gi|134801370|emb	GQLPSYGRLTLPLDPSIDLQLNISFTYGPVILNGDGMDYYESPLLDSGWL	450
German_dog_gi|693948|emb|X8500	GQLPSYGRLTLPLDPSIDLQLNISFTYGPVILNGDGMDYYESPLSDSGWL	450
African Lion (JN812975)	RQLPSYGRLTLPIDPSIDLQLNMSFTYGPVILNGDGMDYYDSPLLDSGWL	450
Lion 1 (MN335911)	RQLPSYGRLTLPIDPSIDLQLNISFTYGPVILNGDGMDYYDSPLLDSGWL	450
Hyena 2 (MN335910)	RQLPSYGRLTLPIDPSIDLQLNISFTYGPVILNGDGMDYYDSPLLDSGWL	450
Spotted Hyena (KJ415364)	--	450
Bat_Eared_Fox (MN335908)	RQLPSYGRLTLPIDPSIDLQLNISFTYGPVILNGDGMDYYDSPLLDSGWL	450
Bat Eared Fox (KC916716)	RQLPSYGRLTLPIDPSIDLQLNISFTYGPVILNGDGMDYYDSPLLDSGWL	450
DOG_H.2_1 (MN335909)	RQLPSYGRLTLPIDPSIDLQLNISFTYGPVILNGDGMDYYDSPLLDSGWL	450
Domestic dog (JN812976)	RQLPSYGRLTLPIDPSIDLQLNISFTYGPVILNGDGMDYYDSPLLDSGWL	450
Hyena 1 (MN335912)	RPLPSYGRLTLPIDPSIDLQLNISFTYGPVILNGDGMDYYESPLLDSGWL	450
Monkey(CYN07-dV(AB687720.2)	GQLPSYGRLTLPLDPSIDLQLNISFTYGPVILNGYGMDYYESPLLDSGWL	450
A75/17 (AF164967.1)	GQLPSYGRLTLPLDPSIDLQLNISFTYGPVILNGDGMDYYESPLLDSGWL	450
Dog_ChinaB_gi|212656655|gb|ACJ	GQLPSYGRLTLPLDPSIDLQLNISFTYGPVILNGDGMDYYESPLLDSGWL	450
German_ferret_gi|693949|emb|X8	GQLPSYGRLILPLDPSIDLQLNISFTYGPVILNGDGMDYYESPLLDSGWL	450
south_african_dog_gi|239949465	GQLPSYGRLTLPLDASIDLQLNISFTYGPVILNGDGMDYYESPLLDSGWL	450
Dog_South_Africa_c_gi|23994946	GQLPSYGRLTLPLDASIDLQLNISFTYGPVILNGEGMDYYESPLLESGWL	450

```
Japanease_dogstrain_007Lmgi|78      VQLPSYGRLTLPLDASIDLQLNISFTYGPVILNGDGMDYYENPLLDSGWL 450
Onderstepoort_gb|AF378705.1|        RQLPSYGRLTLPLDASVDLQLNISFTYGPVILNGDGMDYYESPLLNSGWL 450
Convac_vaccine_gi|517242|emb|Z      RQLPSYGRLTLPLDASVDLQLNISFTYGPVILNGDGMDYYESPLLNSGWL 450
Phoca/Caspian/2007/HM046486         GQLPSYGRLTLPLDASVDLQLNISFTYGPVILNGDGMDYYESPLLNSGWL 450
PDV/ USA/ 2006/HQ007902.1           KRLPSYGRLTLSLDVSTDLSINVSVAQGPIILNGDGMDYYEGTLLNSGWL 450
```

```
Rockborn-Candurgb|GU266280.1|_      TIPPKNGTVLGLINKASRGDQFTVIPHVLTFAPRESSGNCYLPIQTFQIM 500
lesser_panda_gi|5870827|gb|AAD      TIPPKNGTVLGLINKASRGDQFTVIPHVLTFAPRESSGNCYLPIQTFQIM 500
Vanguard_vaccine_gi|239949423|      TIPPKNGTVLGLINKASRGDQFTVIPHVLTFAPRESSGNCYLPIQTFQIM 500
Vacc-P_gi|224579344|gb|FJ70523      TIPPKNGTVLGLINKASRGDQFTVIPHVLTFAPRESSGNCYLPIQTFQIM 500
Dog_argentina_gi|134801370|emb      TIPPKNGTVLGLINKASRGDQFTVIPHVLTFAPRESGGNCYLPIQTSQIM 500
German_dog_gi|693948|emb|X8500      TIPPRNGTVLGLINKASRGDQFTVIPHVLTFAPRESSGNCYLPIQTSQIM 500

African Lion (JN812975)             TIPPKNGTVLGLINKASRGDQFTVIPHVLTFAPRESSGNCYLPIQTSQIM 500
Lion 1 (MN335911)                   TIPPKNGTVLGLINKASRGDQFTVIPHVLTFAPRESSGNCYLPIQTSQIM 500
Hyena 2 (MN335910)                  TIPPKNGTVLGLINKASRGDQFTVIPHVLTFAPRESSGNCYLPIQTSQIM 500
Spotted Hyena (KJ415364)            -------------------------------------GNCYLPIQTSQIM 500
Bat_Eared_Fox (MN335908)            TIPPKNGTVLGLINKASRGDQFTVIPHVLTFAPRESSGNCYLPIQTSQIM 500
Bat Eared fox (KC916716)            TIPPKNGTVLGLINKASRGDQFTVIPHVLTFAPRESSGNCYLPIQTSQIM 500
DOG_H.2_1 (MN335909)                TIPPKNGTVLGLINKASRGDQFTVIPHVLTFAPRESSGKCYLPIQTSQIM 500
Domestic dog (JN812976)             TIPPKNGTVLGLINKASRGDQFTVIPHVLTFAPRESSGKCYLPIQTSQIM 500
Hyena 1 (MN335912)                  TIPPKNGTVLGLINKASRGDQFTVIPHVLTFAPRESSGNCYLPIQTSQIM 500
Monkey(CYN07-dV(AB687720.2)         TIPPKNGTVLGLINKASRGDQFTVTPHVLTFAPRESSGNCYLPIQTSQIM 500

A75/17 (AF164967.1)                 TIPPKNGTVLGLINKASRGDQFTVIPHVLTFAPRESSGNCYLPIQTSQIM 500
Dog_ChinaB_gi|212656655|gb|ACJ      TIPPKNGTVLGLINKASRGDQFTVTPHVLTFAPRESSGNCYLPIQTSQIM 500
German_ferret_gi|693949|emb|X8      TIPPRNGTILGMINKASRGDQFTVTPHVLTFAPRESSGNCYLPIQTSQIM 500
south_african_dog_gi|239949465      TIPPKNGTILGLINKASRGDQFTVIPHVLTFSPRESSGSCYLPIQTSQIM 500
Dog_South_Africa_c_gi|23994946      TIPPKNGTILGLINKASRGDQFTVIPHVLTFAPRGSSGSCYLPIQTSQIM 500
Japanease_dogstrain_007Lmgi|78      TIPPKNGTILGLINKASRGDQFTVTPHVLTFAPRESSGNCYLPIQTSQIM 500
```

Onderstepoort_gb|AF378705.1|	TIPPKNGTIVGLINKAGRGDQFTVLPHVLTFAPWESSGNCYLPIQTSQII	500
Convac_vaccine_gi|517242|emb|Z	TIPPKNGTILGLINKAGRGDQFTVIPHVLTFAPMESSGNCYLPIQTSQII	500
Phoca/Caspian/2007/HM046486	TIPPKNGTILGLINKASRGDQFTVIPQVLTFAPRESCGNCYLPIQTSQII	500
PDV/ USA/ 2006/HQ007902.1	TIPPKNGTILGLINQASKGDQFIVTPHILTFAPRESNTDCHLPIQTSQIQ	500

Rockborn-Candurgb|GU266280.1|_	DKDVLTESNLVVLPTQNFRYVIATYDISRDDHAIVYYVYDPIRTISFTYP	550
lesser_panda_gi|5870827|gb|AAD	DKDVLTESNLVVLPTQNFRYVIATYDISRDDHAIVYYVYDPIRTISFTYP	550
Vanguard_vaccine_gi|239949423|	DKDVLTESNLVVLPTQKFRYVIATYDISRDDHAIVYYVYDPIRKISFTYP	550
Vacc-P_gi|224579344|gb|FJ70523	DKDVLTESNLVVLPTQNFRYVIAKYDISRDDHAIVYYVYDPIRKISFTYP	550
Dog_argentina_gi|134801370|emb	DKDVLAESNLVVLPTQNFRYVIATYDISRDDHAIVYYVYDPIRTISYTYP	550
German_dog_gi|693948|emb|X8500	DKDVLTESNLVVLPTQNFRYVIATYDISRGDHAIVYYVYDPIRTISYTYP	550
African Lion (JN812975)	DKDVLTESNLVVLPTQNFIYVIATYDVSRDDHAIVYYVYDPIRTISYTHP	550
Lion 1 (MN335911)	DKDVLTESNLVVLPTQNFIYVIATYDVSRDDHAIVYYVYDPIRTISYTHP	550
Hyena 2 (MN335910)	DKDVLTESNLVVLPTQNFIYVIATYDVSRDDHAIVYYVYDPIRTISYTHP	550
Spotted Hyena (KJ415364)	DKDVLTESNLVVLPTQNFIYVIATYDVSRDDHAIVYYVYDPIRTISYTHP	
Bat_Eared_Fox (MN335908)	DKDVLTESNLVVLPTQNFRYVIATYDVSRDDHAIVYYVYDIPRTISYTYP	550
Bat Eared Fox (KC916716)	DKDVLTESNLVVLPTQNFRYVIATYDVSRDDHAIVYYVYDPIRTISYTHP	550
DOG_H.2_1 (MN335909)	DKDVLTESNLVVLPTQNFRYVIATYDVSRDDHAIVYYVYDPIRTISYTYP	550
Domestic dog (JN812976)	DKDVLTESNLVVLPTQNFRYVIATYGVSRDDHAIVYYVYDPIRAISYTYP	550
Hyena 1 (MN335912)	DKDVLTESNLVVLPTQNFRYVIATYDISRGDHAIVYYVYDPIRAISYTYP	550
Monkey(CYN07-dV(AB687720.2)	DKDVLTESNLVVLPTQNFRYVIATYDISRGDHANVYYVYDPFRTISYTYP	550
A75/17 (AF164967.1)	DKDVLTESNLVVLPTQNFRYVIATYDISRGDHAIVYYVYDPIRAISYTYP	550
Dog_ChinaB_gi|212656655|gb|ACJ	DKDVLTESNLVMLPTQNFRYVIATYDISRGDHAIVYYVYDPIRTISYTYP	550
German_ferret_gi|693949|emb|X8	DKDVLIESNLVVLPTQNFRYVIATYDISRDDHAIVYYVYDPIRTISYMYP	550
south_african_dog_gi|239949465	DKDVLTESNLVVLPTQNFRYVVATYDISRNDHAIVYYVYDPIRTISYTYP	550
Dog_South_Africa_c_gi|23994946	DKDVLTESNLVVLPTQNFRYVVATYDISRNDHAIVYYVYDPIRTISYTYP	550
Japanease_dogstrain_007Lmgi|78	DKDVLTESNLVVLPTQNFRYVVATYDISRENHAIVYYVYDPIRTISYTYP	550

```
Onderstepoort_gb|AF378705.1|       DRDVLIESNIVVLPTQSFRYVIATYDISRSDHAIVYYVYDPIRTISYTHP 550
Convac_vaccine_gi|517242|emb|Z     DRDVLIESNLVVLPTQSFRYVIATYDISRSDHAIVYYVYDPIRTISYTHP 550
Phoca/Caspian/2007/HM046486        DRDVLIESNVVVLPTQSFRYVIATYDISRNDHAIVYYVYDPIRTISYTHP 550
PDV/ USA/ 2006/HQ007902.1          DDDVLLESNLVVLPTQSFEYVVATYDVSRSDHAIVYYVYDPARTVSYTYP 550
```

Rockborn-Candurgb|GU266280.1|_	FRLTTKGRPDFLRIECFVWDDDLWCHQFYRFEADITNFTTSVENLVRIRF	600
lesser_panda_gi|5870827|gb|AAD	FQLTTKGRPDFLRIECFVWDDDLWCHQFYRFEADITNFTTSVENLVRIRF	600
Vanguard_vaccine_gi|239949423|	FRLTTKGRPDFLRIECFVWDDDLWCHQFYRFEADITDSTTSVENLVRIRF	600
Vacc-P_gi|224579344|gb|FJ70523	FRLTTKGRPDFLRIECFVWDDDLWCHQFYRFEADITNFTTSVENLVRIRF	600
Dog_argentina_gi|134801370|emb	FRLTTKGRPDFLRIECFVWDDDLWCHQFYRFEADITNSTTSIED------	600
German_dog_gi|693948|emb|X8500	FRLTTKGRPDFLRIECFVWDDDLWCHQFYRFEADITNSTTSVENLVRIRF	600
African Lion (JN812975)	FRLTTKGRPDFLRIECFVWDDDLWCHQSYRFEADVTNSTTSVENLVCIRF	600
Lion 1 (MN335911)	FRLTTKGRPDFLRIECFVWDDDLWCHQFYRFEADVTNSTTSVENLVCIRF	600
Hyena 2 (MN335910)	FRLTTKGRPDFLRIECFVWDDDLWCHQFYRFEADVTNSTTSVENLVCIRF	600
Spotted Hyena (KJ415364)	FRLTTKGRPDFLRIECFVWDDDVWCHQFYRFEADVTNSTTSVENLVCIRF	600
Bat_Eared_Fox (MN335908)	FRLTTKGRPDFLRIECFVWDDDLWCHQFYRFEADVTNSTTSVENLVCIRF	600
Bat Eared Fox (KC916716)	FRLTTKGRPDFLRIECFVWDDDLWCHQFYRFEADVTNSTTSVENLVCIRF	600
DOG_H.2_1 (MN335909)	FRLTTKR---	600
Domestic dog (JN812976)	FRLTTKGRPDFLRIECFVWDDDLWCHQFYRFEADVTNSTTSVENLVCIRF	600
Hyena 1 (MN335912)	FRLTTKGRPDFLRIECFVWDDDLWCHQFYRFEADSTNSTTSVENLVRIRF	600
Monkey(CYN07-dV(AB687720.2)	FRLTTKGRPDFLRIECFVWDDDLWCHQFYRFEANITNSTTSVENLVRIRF	600
A75/17 (AF164967.1)	FRLTTKGRPDFLRIECFVWDDDLWCHQFYRFEADSTNSTTSVENLVRIRF	600
Dog_ChinaB_gi|212656655|gb|ACJ	FRLTTKGRPDFLRIECFVWDDDLWCHQFYRFEANITNSTTSVENLVRIRF	600
German_ferret_gi|693949|emb|X8	FRLTTKGRPDFLRIECFVWDNDLWCHQFYRFEADITNSTSSVENLVRIRF	600
south_african_dog_gi|239949465	FRLTTKGRPDFLRIECFVWDDDLWCHQFYRFETDITNSTTSVENLVRIRF	600
Dog_South_Africa_c_gi|23994946	FRLTTKGKPDFLRIECFVWDDDLWCHQFYRFEADITNSTTSVENLVRIRF	600
Japanease_dogstrain_007Lmgi|78	FRLTTKGRPDFLRIECFVWDDDLWCHQFYRFESDITNSTTSVEDLVRIRF	600

```
Onderstepoort_gb|AF378705.1|        FRLTTKGRPDFLRIECFVWDDNLWCHQFYRFEADIANSTTSVENLVRIRF 600

Phoca/Caspian/2007/ADG96017.1      FLRIECFVWDDNLWCHQFYRYEANIANSTTSVENLVRIRFSCNRSNP   600

PDV/ USA/ 2006/HQ007902.1           FRLRTKGRPDILRIECFVWDGHLWCHQFYRFQLDATNSTSVVENLIRIRF 600
```
